# Rational Design of a Phage Cocktail for Effective Control of Multidrug-Resistant Uropathogenic *Escherichia coli* from Hospitalized Patients

**DOI:** 10.3390/antibiotics15090870

**Published:** 2026-09-06

**Authors:** Patiphan Khunti, Panupon Mongkolkarvin, Songphon Buddhasiri, Joe Pogliano, Poochit Nonejuie, Parameth Thiennimitr, Vorrapon Chaikeeratisak

**Affiliations:** 1 Department of Biochemistry, Faculty of Science, Chulalongkorn University, Bangkok 10330, Thailand; 2 Department of Microbiology, Faculty of Medicine, Chiang Mai University, Chiang Mai 50200, Thailand; 3 Veterinary Public Health and Food Safety Centre for Asia Pacific, Faculty of Veterinary Medicine, Chiang Mai University, Chiang Mai 50100, Thailand; 4 Department of Molecular Biology, University of California San Diego, La Jolla, CA 92093, USA; 5 Institute of Molecular Biosciences, Mahidol University, Nakhon Pathom 73170, Thailand; 6 Center of Excellence in Microbial Diversity and Sustainable Utilization, Chiang Mai University, Chiang Mai 50200, Thailand; 7 Center of Excellence for Molecular Biology and Genomics of Shrimp, Faculty of Science, Chulalongkorn University, Bangkok 10330, Thailand

**Keywords:** bacteriophage, phage, phage therapy, UPEC, MDR, MDR-UPEC

## Abstract

Background: The emergence of multidrug-resistant (MDR) uropathogenic *Escherichia coli* (UPEC) poses a significant public health challenge, and alternative treatments are urgently needed. Methods: Here, we identified clinical MDR-UPEC strains AT82 and AT84 collected from hospitalized patients that display extensive antimicrobial resistance at both genetic and phenotypic levels. Due to their high resistance profile, we systematically customized a phage cocktail from our coliphage library using hierarchical clustering based on host specificity and candidate selection through bacterial suppression profiles. Results: This pipeline yielded four lytic coliphages, designated Phi25-4, Phi25-6, Phi50-4, and Killian. Their genomes are relatively large ranging from 112–169 kbp and cluster into two distinct lineages comprising two closely related groups: Phi25-4/Phi50-4 and Phi25-6/Killian. Although each phage exhibited potent antibacterial activity, none alone sustained bacterial suppression during prolonged treatment. To overcome this limitation, we systematically compared the antibacterial activity of all possible phage combinations. Conclusions: The four-phage cocktail outperformed all two- or three-phage formulations, sustaining significant growth inhibition of AT82 and AT84 for up to 16 h and reducing area under the curve by more than 80% relative to controls. Cocktail potency was dose-dependent, with lower phage doses yielding the least viable cells at 48 h. Additionally, this cocktail exerted prophylactic action, significantly reducing UPEC invasion by several orders of magnitude, while the phage cocktail alone induced minimal proinflammatory cytokine responses in human bladder epithelium. Together, these findings provide an effective phage cocktail and a complementary framework for cocktail design against urinary tract infections caused by MDR bacteria.

## 1. Introduction

Urinary tract infections (UTIs) are among the most common bacterial infections worldwide and continue to cause a major burden on healthcare systems. They affect millions of individuals each year, with women being disproportionately impacted due to anatomical and physiological factors [1,2,3]. Uncomplicated UTIs are caused mainly by uropathogenic *Escherichia coli* (UPEC), a specialized lineage of *E. coli* characterized by distinct virulence factors that facilitate adhesion, invasion, and intracellular persistence within human bladder epithelial cells [1,4,5,6,7]. Although UTIs are often treated with short courses of antibiotics [8,9], therapeutic outcomes are increasingly threatened by the global rise in multidrug-resistant (MDR) UPEC strains. Over recent decades, widespread and repeated antibiotic use has selected for UPEC populations resistant to multiple clinically important drug classes, including aminoglycosides, β-lactams, carbapenems, and fluoroquinolones [6,10,11,12,13,14,15]. As a result, recurrent and chronic infections are becoming more frequent, leading to higher rates of treatment failure, reinfection, and increased healthcare costs [1,16,17].

Given these clinical challenges, there is a strong need for alternative therapeutic strategies that act independently of conventional antibiotic mechanisms. One approach receiving renewed attention is bacteriophage (phage) therapy. Phages are viruses that infect and kill bacteria with remarkable specificity, enabling targeted elimination of pathogens while preserving beneficial host microbiota [18,19]. Because they replicate at the site of infection and can continue to amplify as long as susceptible bacteria are present, phages offer a dynamic mode of antibacterial activity that differs fundamentally from fixed-dose antibiotics. However, the use of a single lytic phage often leads to the emergence of phage-resistant bacterial variants, which can rapidly repopulate after initial clearance [20]. To overcome this limitation, the use of multiple lytic phages in combination, commonly referred to as a phage cocktail, has emerged as a promising approach. Combining phages that target the same bacterial host in different mechanisms can broaden host coverage and reduce the likelihood of bacteria resistance [21,22]. The challenge lies in choosing compatible phages that are both effective and sufficiently diverse to provide broad antibacterial activity. Therefore, the development of an effective phage cocktail composed of well-characterized and genetically diverse lytic phages may provide an alternative approach for controlling MDR-UPEC infections. However, before a phage cocktail can be considered for therapeutic application, the individual phages should be carefully characterized in terms of their host range, infection dynamics, genomic features, and antibacterial activity. In addition, the activity of the final cocktail formulation should be evaluated under different treatment conditions and in a model that more closely reflects the interaction between UPEC and human bladder epithelial cells.

In this study, we aimed to isolate and characterize lytic bacteriophages active against UPEC and to select a combination of phages with complementary properties for the development of an effective phage cocktail. The selected phages were examined for their biological characteristics, genomic safety, and antibacterial activity against MDR-UPEC strains. We then evaluated the ability of different phage combinations to suppress bacterial growth and assessed the effect of phage dose on antibacterial efficacy and bacterial regrowth. Finally, the selected cocktail was tested in a human bladder epithelial cell model to determine whether it could reduce UPEC invasion and influence infection-associated inflammatory responses. These experiments were performed to assess whether a rationally selected phage cocktail could provide a promising alternative or complementary strategy for the control of MDR UPEC-associated UTIs.

## 2. Results

### 2.1. Clinical UPEC Isolates Exhibit Diverse Resistance Profiles, with AT82 and AT84 Identified as Multidrug-Resistant Bacteria

**Figure 1:**
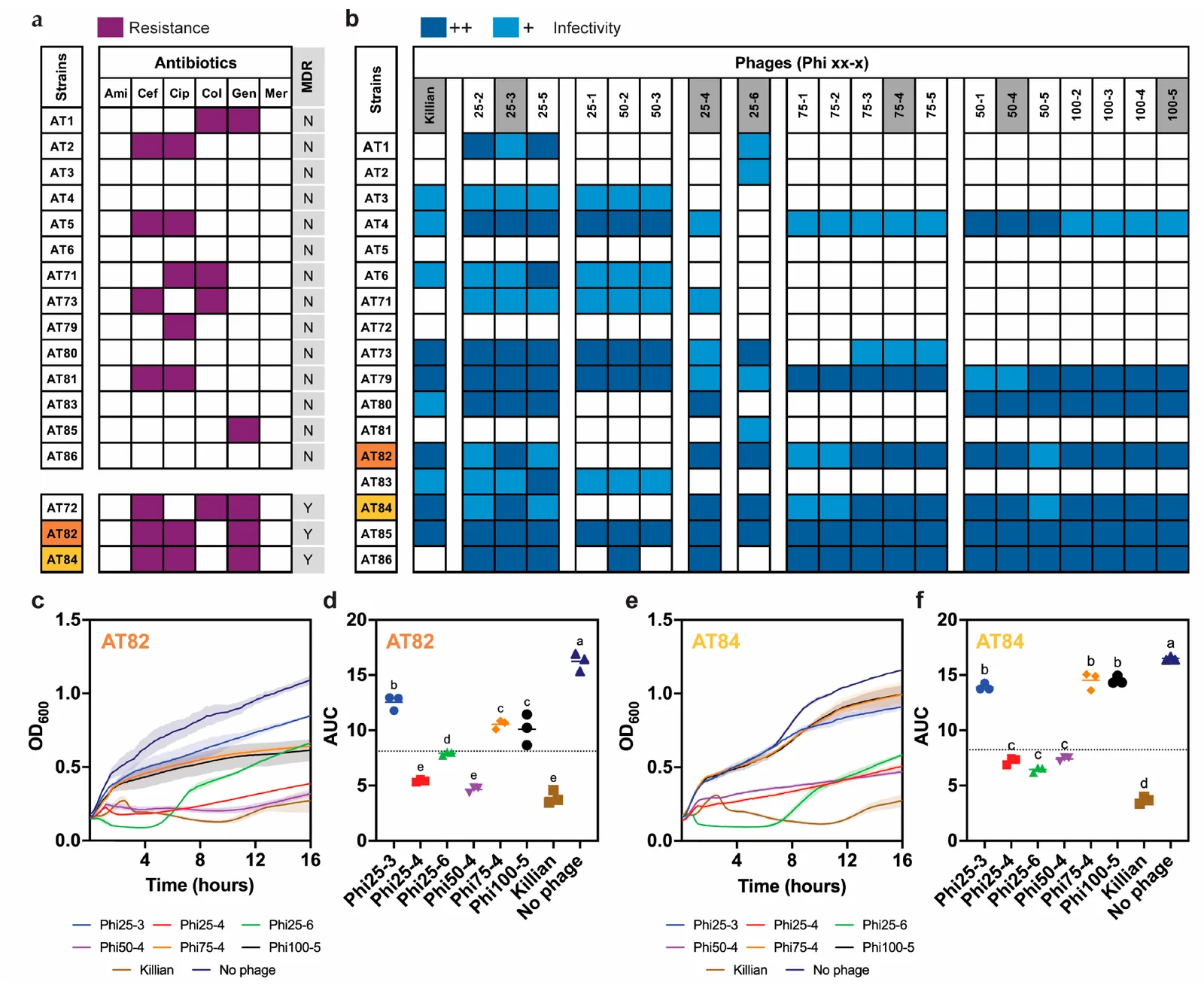
Antibiotic resistance profile, host range, and killing activity of the phage library against clinical UPEC isolates. (**a**) Antibiotic resistance patterns of 17 clinical *E. coli* isolates against amikacin (Ami), ceftriaxone (Cef), ciprofloxacin (Cip), colistin (Col), gentamicin (Gen), and meropenem (Mer). Purple squares indicate resistance. MDR = multidrug resistance (yes: Y or no: N). (**b**) Host range of the phage library across all clinical UPEC isolates. Infection outcomes were scored as strong clearing (++), intermediate clearing (+), or no infection, and are shown as dark blue, light blue, and white squares, respectively. The phages were hierarchically clustered based on host spectrum similarity using the Jaccard metric. Representative phages from each cluster were selected for downstream characterization, as indicated by grey labels on their names. (**c**,**e**) Representative killing curves against MDR isolates AT82 (C) and AT84 (E). OD_600_ was measured over 16 h to assess bacterial growth in the presence of individual phages. Data are presented as the mean ± standard deviations (SD) from at least three independent experiments, with solid lines representing the mean and shaded regions indicating SD. (**d**,**f**) Area-under-the-curve (AUC) analysis corresponding to panels C and E. Lower AUC values reflect stronger overall killing. Phages achieving a reduction of >50% in AUC compared to the control were selected as candidates for cocktail formulation. Error bars represent the mean ± sd. of three biological replicates. Different letters indicate significant differences (*p* < 0.05, one-way ANOVA with multiple comparisons correction).

A panel of 17 clinical UPEC isolates (AT followed by numbers; Table S1) was collected following the collection from urine culture of patients admitted at Maharaj Nakorn Chiang Mai Hospital (MNCMH), Chiang Mai, Thailand. To evaluate their antibiotic susceptibility status, minimum inhibitory concentrations (MICs) were determined against six clinically relevant antibiotics: amikacin, ceftriaxone, ciprofloxacin, colistin, gentamicin, and meropenem (Figure 1a and Table S2). These clinical isolates exhibited a diverse range of resistance phenotypes, varying from susceptibility to all antibiotics to resistance to multiple antibiotics. Specifically, clinical isolates AT72, AT82, and AT84 were resistant to multiple antibiotic classes, confirming their multidrug-resistant (MDR) phenotypes. While UPEC strains AT82 and AT84 shared similar resistance profiles, exhibiting resistance to ceftriaxone, ciprofloxacin, and gentamicin, UPEC AT72 showed strong resistance to colistin, a last-line antibiotic for Gram-negative pathogens, but remained susceptible to ciprofloxacin (Figure 1a and Table S2; highlighted in grey).

The two representative MDR-UPEC isolates AT82 and AT84 were whole-genome sequenced, reporting their genome sizes and predicted gene content (Accession numbers; AT82: JBPSNA000000000 and AT84: JBPSNB000000000) [23]. To elucidate the molecular basis of their resistance, we investigated the presence of genes associated with the observed resistance profiles. As summarized in Table 1 and Table 2, both genomes harbored multiple resistance genes, including *blaCTX-M-55*, *blaTEM-1B*, which together underlie their broad β-lactam resistance. Genes encoding aminoglycoside-modifying enzymes (*aac(3)-IId*) and quinolone resistance determinants (*qnrS1*) were also detected. The WGS-predicted resistance phenotypes (Table 2) were highly consistent with the observed MIC data (Figure 1a), confirming that AT82 and AT84 possess complex, genome-encoded resistance mechanisms. Overall, these results identified the clinical MDR-UPEC isolates form a diverse and clinically relevant collection. Based on their high resistance levels supported by both genomic and phenotypic data, UPEC AT82 and AT84 were selected as representative bacteria for downstream experiments.

### 2.2. Host Range–Based Clustering and Rational Phage Selection for MDR-UPEC AT82 and AT84

In the era of escalating antibiotic resistance, effective alternative strategies are urgently needed. The MDR-UPEC AT82 and AT84 exhibit high-level resistance to three clinically relevant antibiotics, highlighting the urgent need for therapeutic solutions. To identify potential phage candidates, we first constructed a phage library targeting UPEC isolates through enrichment and isolation using UPEC UTI89 as an enrichment host (Figure S1) from hospital wastewater [22]. We further purified them through Percoll gradient centrifugation to differentiate them based on particle sizes, leading to the isolation of new phages. Since coliphage Killian has been investigated for its therapeutic potential against UPEC isolates previously [24], it was also included in this phage library. The lytic potential of newly isolated phages and Killian was then assessed against all clinical isolates using a spot test. Infection outcomes were recorded as high, immediate, or no lysis, and visualized in a host range heatmap. Since we initially had no indication of whether the isolated phages were identical, phage selection therefore relied solely on the host spectrum. To identify groups of presumably related phages, we performed hierarchical clustering based on host specificity similarity (see Section 4), which resulted in seven clusters according to their infection profiles across the clinical UPEC panel (Figure 1b). Within each cluster, phages displayed similar host range, suggesting a close relationship. Notably, Killian, Phi25-4, and Phi25-6, formed unique single-member clusters, reflecting their distinct host spectra. Together, this clustering approach facilitated phage selection within the library and highlighted their strain-specific infectivity patterns.

To select potential phages displaying lytic properties against MDR-UPEC AT82 and AT84 while avoiding redundancy and ensuring broad host coverage, we established the following selection criteria: candidates (1) must demonstrate highly efficient lysis (Figure 1b; dark blue panel) and (2) must be capable of infecting both MDR-UPEC AT82 and AT84 (Figure 1a; orange and yellow panels). In most cases, only a single representative per cluster was retained, including Killian, Phi25-3, Phi25-4, Phi25-6, and Phi75-4 (Figure 1b; highlighted phages in grey). However, the seventh cluster (the rightmost group in Figure 1b) included two phage populations, Phi50-X and Phi100-X, which exhibited markedly different plaque morphologies despite belonging to the same cluster. Phi50-X produced relatively small plaques, whereas Phi100-X formed large plaques. Because plaque size reflects differences in replication kinetics or lytic potency, representatives (Phi50-4 and Phi100-5) from the group were retained as independent candidates. All representative phages (Figure 1b; highlighted phages in grey) were subsequently subjected to further characterization.

### 2.3. Phi25-4, Phi25-6, Phi50-4, and Killian Are Key Candidates for Cocktail Formulation Due to Their Robust Lytic Activity

To identify promising candidates for cocktail formulation, we evaluated the killing kinetics of selected phages individually against MDR-UPEC AT82 and AT84 by monitoring bacterial cell density over a 16-h treatment at a multiplicity of infection (MOI) of 1 (Figure 1c,e; AT82 and AT84)**.** The result showed that several phages, including Phi25-4, Phi25-6, Phi50-4, and Killian, sharply reduced bacterial density in both UPEC strains during the initial hours post infection, indicating rapid adsorption and efficient lytic activity. Even though these phages effectively suppressed bacterial growth at early time points, the bacteria resumed growth as the optical density gradually increased at later time points. This suggests the emergence of phage-resistant subpopulations or incomplete suppression of surviving cells, indicating bacterial adaptation under selective pressure. In contrast, phages Phi25-3, Phi75-4, and Phi100-5 exerted minimal suppressive effects on bacterial growth (Figure 1c,e).

To quantitatively define lytic performance, we employed area under the curve (AUC) analysis of the bacterial growth profiles, as previously described [25]. Based on our selection criterion, phages that markedly suppressed bacterial growth leading to the reduction in the AUC by at least 50% relative to the no-phage control were considered as exhibiting strong lytic activity (Figure 1d,f; AT82 and AT84). Therefore, a subset of phages, including Phi25-4, Phi25-6, Phi50-4, and Killian (Figure 1d,f; red, green, purple, and brown), was selected as the core candidate set for cocktail formulation, due to their strong lytic profiles against MDR-UPEC strains. This AUC-based evaluation provided a quantitative benchmark for identifying robustly lytic and therapeutically promising phages from our diverse isolate collection.

### 2.4. Morphological and Biological Characterization of Phage Candidates

**Figure 2:**
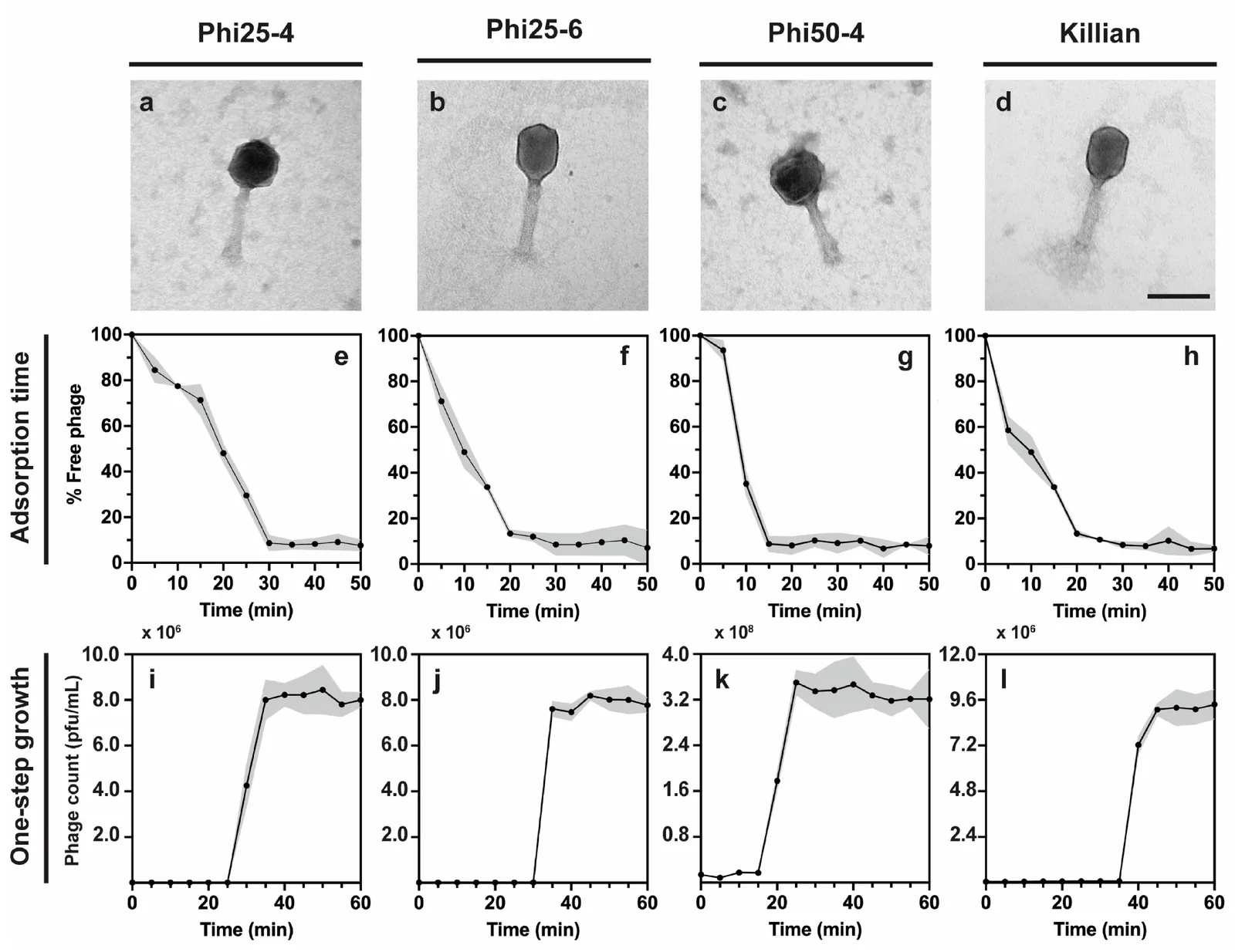
Morphological and biological properties of selected phages. (**a**–**d**) Transmission electron micrographs (TEM) of phages Phi25-4, Phi25-6, Phi50-4, and Killian. All phages display a contractile tail and *Myoviridae*-like morphology, with Phi25-4 and Phi50-4 showing icosahedral capsids and Phi25-6 and Killian displaying elongated capsids. Scale bar = 100 nm. (**e**–**h**) Adsorption kinetics on MDR-UPEC AT82. The percentage of unadsorbed (free) phage particles was measured over 50 min. All four candidates adsorbed to the host, with >80% attachment occurring within 15–30 min. (**i**–**l**) One-step growth curves of the four phages on AT82. Latent periods ranged from ~15–35 min depending on the phage type, followed by a sharp increase in phage titer representing progeny release. Data are presented as the mean ± standard deviations (SD) from at least three independent experiments, with solid lines representing the mean and shaded regions indicating SD.

Transmission electron microscopy (TEM) revealed that all four phages, Phi25-4, Phi25-6, Phi50-4, and Killian, possessed either icosahedral heads (Phi25-4 and Phi50-4) or elongated capsids (Phi25-6 and Killian), together with a contractile tail. Based on their phenotype, they possess myovirus morphology (Figure 2a–d). To better understand their infection dynamics against MDR-UPEC, we conducted adsorption and one-step growth assays to quantify key kinetic parameters, including adsorption rate, latent period, and burst size. These parameters are essential indicators of phage potency, as rapid attachment and high progeny production generally correlate with more effective bacterial clearance, whereas slow adsorption or limited progeny release can compromise infection efficiency, particularly in rapidly dividing bacterial populations [26].

Since these phage candidates are aimed to apply against the MDR-UPEC strains, we decided to investigate the phage potency in MDR-UPEC AT82 as a bacterial host model. The adsorption assays revealed that more than 80% of phage particles in all four candidates were able to attach to host cells within 15–30 min, depending on the phage type. Among them, Phi50-4 displayed the most rapid adsorption rate compared with the others, while Phi25-4 showed the lowest adsorption efficiency (Figure 2e–h). One-step growth curve analyses further demonstrated distinct replication cycles among all phage candidates. These phages underwent intracellular replication ranging from approximately 15 to 35 min, with the shortest period observed in Phi50-4 and the longest in Killian. Following the latent phases, all candidates exhibited a sharp rise in phage titers, indicating strong lytic capacities after the completion of the reproduction cycle leading to phage releases (Figure 2i–l). The burst sizes of Phi25-4, Phi25-6, Phi50-4, and Killian were calculated at approximately 129 ± 3.89, 121 ± 4.32, 153 ± 2.88, and 134 ± 6.26 particles per infected cell, respectively, indicating their efficient replication cycles. These findings reveal considerable variation in infection kinetics among the four key candidates, offering insights into their phage-host interactions and their therapeutic potential.

### 2.5. Genome Analysis Reveals the Therapeutic Safety and Genetic Relationships Among Phage Candidates

**Figure 3:**
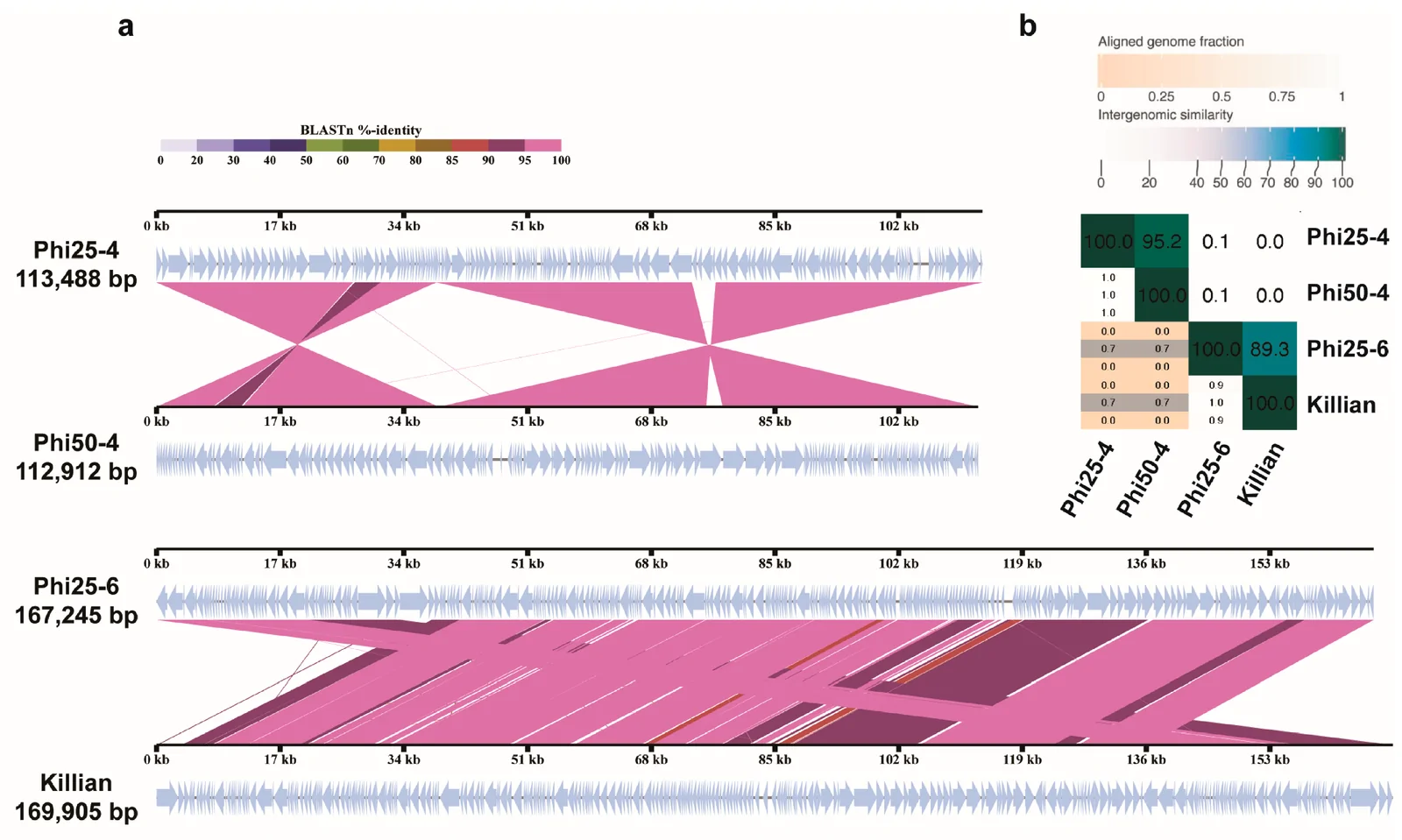
Comparative genomic analysis of the four selected MDR-UPEC-targeting phages. (**a**) Whole-genome alignments of Phi25-4, Phi50-4, Phi25-6, and Killian were generated using DiGAlign. Colored blocks indicate regions of nucleotide similarity, with shading corresponding to percent identity. Phi25-4 and Phi50-4 share extensive sequence similarity but differ in genome organization, showing multiple rearrangements and inversions that distinguish them as separate isolates. Phi25-6 and Killian also display large homologous regions yet maintain distinct genomic architectures. No conserved sequence blocks are shared across the two pairs (Phi25-4/Phi50-4 vs. Phi25-6/Killian), revealing two genetically distinct phage lineages. Gene arrows represent annotated open reading frames and their orientation along each genome. (**b**) VIRIDIC intergenomic similarity matrix displaying percent nucleotide similarity (lower right) and fraction of aligned genome (upper left). All four phages fall below the 95% species-level threshold, confirming that each represents a distinct species. Phi25-4 and Phi50-4 show the highest similarity (95.2%), followed by Phi25-6 and Killian (89.3%), while no substantial similarity is observed across the two lineage pairs. Although Phi25-4 and Phi50-4 share >95% nucleotide identity, the alignment reveals multiple inversions and rearrangements in genome orientation, confirming that they are distinct isolates rather than identical phages.

To better understand the genetic background and ensure the safety of our phage candidates, whole-genome sequencing was performed. Genome sequencing is an essential step for characterizing newly isolated phages, as it provides insight into their replication lifestyle and overall suitability for therapeutic application. Importantly, genome analysis also allows the detection of undesirable genetic features, including the presence of integrases or toxin-associated and antibiotic-resistance genes, which could compromise their safety in clinical settings. Sequencing results revealed that Phi25-4 (Accession number: PX393109), Phi25-6 (Accession number: PX393108), Phi50-4 (Accession number: PX393110), and Killian (Accession number: OQ446694) [24] possess double-stranded DNA genomes of approximately 113, 167, 112, and 169 kb, with GC contents of 45.5%, 35.6%, 45.7%, and 35.5%, respectively (Figure 3a and Figure S2). Genome annotation showed a typical organization of phages, with gene modules encoding factors required for reproduction, virion structural assembly, and host–cell lysis. Because none of the genomes encoded integrases or lysogenic-associated genes, toxins, virulence factors, or antimicrobial resistance genes, these data confirm that all isolates exhibit a strictly lytic life cycle and lack genetic elements associated with lysogeny or horizontal gene transfer, rendering them appropriate for therapeutic use.

Phage cocktails composed of genetically diverse phages have been proposed as a promising strategy for cocktail design, as resistance is less likely to emerge when multiple independent mutations are required to confer protection against various phages in the mixture [21]. To explore whether our isolates represent novel species and to assess their genetic relationships, we conducted comparative genomic analyses. Such analyses are valuable, as they enable distinction among related isolates and allow identification of genome rearrangements that would not be captured by sequence similarity alone. VIRIDIC analysis was first conducted to determine the intergenomic similarity of our phage candidates relative to other phages in public databases. The result revealed that all four isolates represent novel species, as all showed intergenomic similarity values below the 95% species-level threshold [27] (Figure S2E). Within our isolate set, Phi25-6 and Killian shared 89.3% similarity, while Phi25-4 and Phi50-4 were more closely related, exhibiting 95.2% similarity (Figure 3b). Further comparison of genome organization confirmed these relationships: each pair (Phi25-4 with Phi50-4, and Phi25-6 with Killian) was closely related, as they shared numerous homologous genes (Figure 3a). Particularly, although Phi25-4 and Phi50-4 exhibited extensive sequence similarity, displaying over 95% intergenomic similarity, their genomes differed in gene organization, with several regions showing rearranged orientation and gene order, indicating that they are distinct isolates rather than identical phages. In contrast, no conserved homologs were shared across groups (between the Phi25-4/Phi50-4 pair and the Phi25-6/Killian pair), highlighting their distant genetic relationship. Together, these findings demonstrate that several isolated phages are genetically diverse, supporting their suitability as candidates for cocktail formulation. Such genetic diversity provides a rational basis for combining multiple phages to minimize the likelihood of resistance development.

### 2.6. Four-Phage Formulations Display Superior Antibacterial Activity Against MDR-UPEC Strains

**Figure 4:**
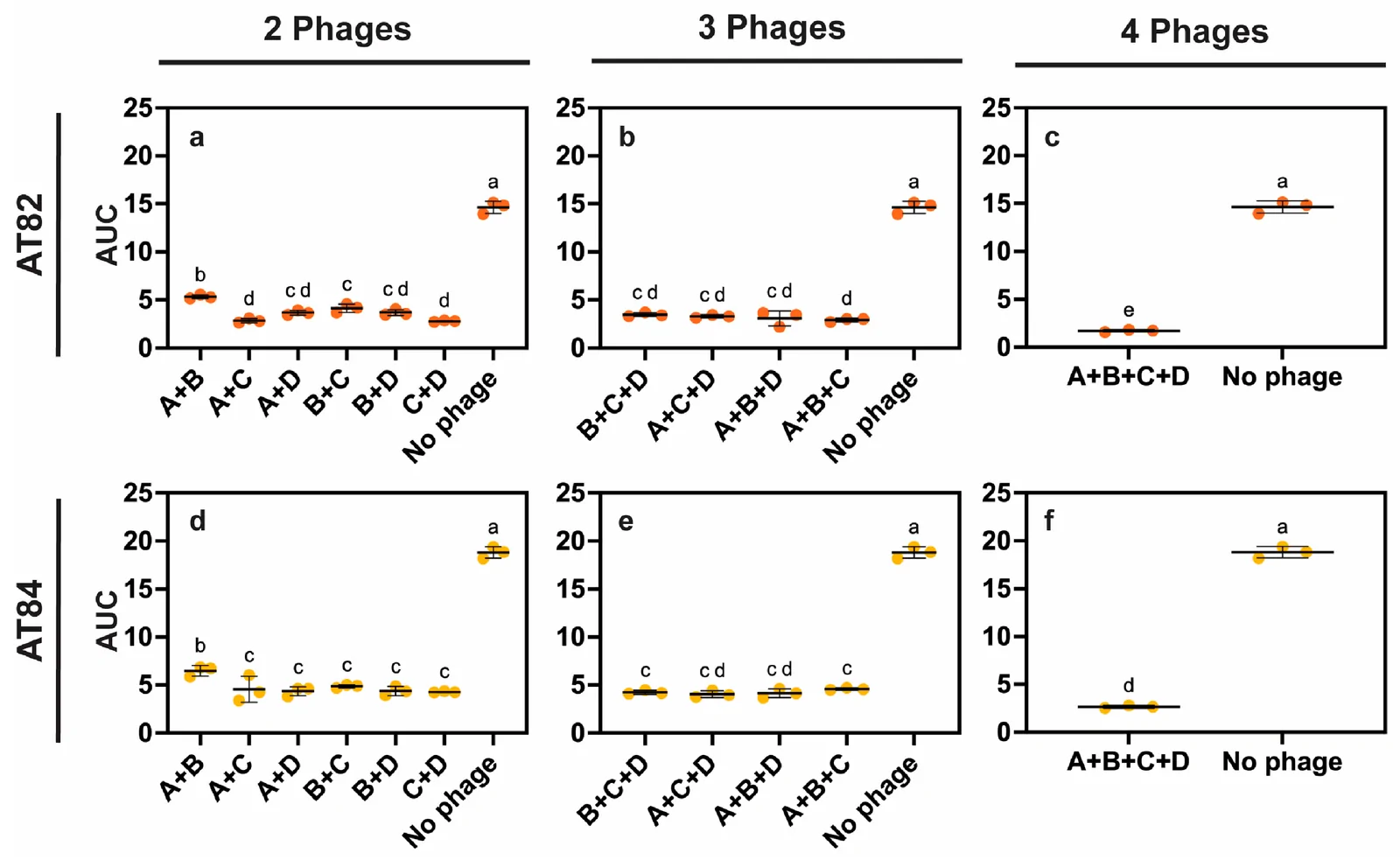
Evaluation of two-, three-, and four-phage combinations against MDR-UPEC strains AT82 and AT84. Area-under-the-curve (AUC) analysis comparing bacterial growth suppression by pairwise (**a**,**d**), triple (**b**,**e**), and four-phage (**c**,**f**) combinations of Phi25-4 (A), Phi25-6 (B), Phi50-4 (C), and Killian (D) against AT82 (**top** row) and AT84 (**bottom** row) over a 16-h incubation period. Each point represents a biological replicate, and horizontal bars show the mean AUC values. The four-phage cocktail (A + B + C + D) produced the greatest reduction in AUC for both strains, indicating sustained inhibition compared with all other treatments and the no-phage control. Treatments that do not share the same letter are significantly different (one-way ANOVA, *p* < 0.05).

To design an effective phage cocktail, we systematically compared the antibacterial activity of all possible phage combinations (two-, three-, and four-phage formulations) composed of Phi25-4 (A), Phi25-6 (B), Phi50-4 (C), and Killian (D) (Figure 4). The efficacy of each formula was assessed against MDR-UPEC strains AT82 and AT84 at a total MOI of 1 by monitoring bacterial cell density (Figure S3) and evaluating AUC (Figure 4) with the criteria described above. The result showed that both double- and triple- phage formulations exhibited only partial inhibitory effects at the initial period of treatment (Figure S3). Almost all combinations in these categories were able to suppress bacterial growth for only around 10 h before bacterial regrowth began, indicating the emergence of resistant subpopulations or incomplete eradication of survivors. Notably, two combinations, Phi25-6 (B) + Phi50-4 (C) and Phi25-4 (A) + Phi25-6 (B) + Phi50-4 (C) (Figure S3; orange line), displayed a distinct suppression pattern, maintaining lower bacterial densities over prolonged incubation periods. In several cases, bacterial density gradually increased at later time points, indicating that other double- and triple- phage formulations can delay bacterial growth but cannot completely eradicate the bacteria (Figure S3). In contrast, the four-phage formulation (all phages: A + B + C + D) provided the most sustained suppression against both MDR-UPEC strains (Figure S3C,F). For AT82, bacterial density remained near baseline throughout the 16-h observation period, with no detectable regrowth (Figure S3C). For AT84, a slight increase in density was observed during the first 1–4 h, likely reflecting early bacterial adaptation or delayed phage adsorption; however, the density rapidly declined thereafter and remained close to baseline for the rest of the treatment (Figure S3F).

AUC-based quantitative analysis further demonstrated the superior efficacy of the four-phage formulation, which reduced the AUC by more than 80% compared with the no-phage control for both MDR-UPEC strains (Figure 4c,f; AT82 and AT84). Our statistical analysis further revealed that this formulation was significantly more effective than all other combinations tested (*p* < 0.0001). Interestingly, a similar suppressive effect was also observed for some particular triple-phage formulations (A + C + D and A + B + D) when tested against AT84 (Figure 4e,f). The consistency of these findings across two distinct MDR-UPEC strains highlights the potency of the four-phage formulation, which was therefore selected as the most effective cocktail for subsequent optimization.

### 2.7. Optimal Phage Doses for Sustained Bacterial Suppression

**Figure 5:**
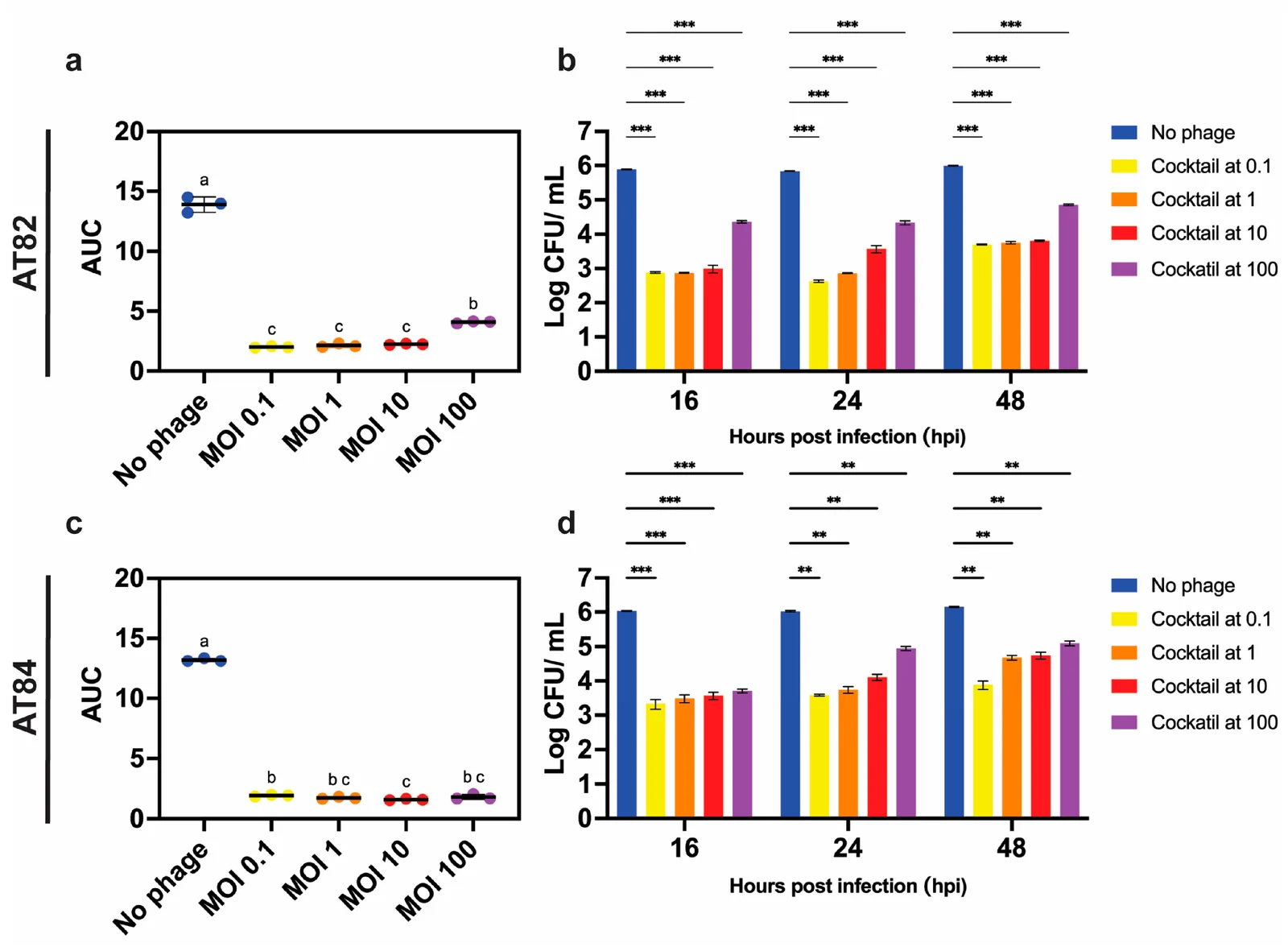
Dose-dependent suppression of MDR-UPEC by the four-phage cocktail. (**a**,**c**) Area-under-the-curve (AUC) analysis of bacterial growth inhibition by the phage cocktail at multiplicities of infection (MOIs) 0.1, 1, 10, and 100 compared with a no-phage control for AT82 (**a**) and AT84 (**c**) over a 16-h infection period. Each point represents a biological replicate, and horizontal bars indicate the mean AUC. Treatments that do not share the same letter differ significantly (*p* < 0.05; one-way ANOVA). (**b**,**d**) Viable bacterial counts (log CFU/mL) for AT82 (**b**) and AT84 (**d**) following cocktail treatment at the indicated MOIs for 16, 24, and 48 h. All cocktail conditions significantly reduced CFU compared with the no-phage control at each time point, with differences in durability depending on the strain and MOI. Data represent mean ± SD from at least three independent experiments. Different lowercase letters indicate statistically significant differences among treatment groups, whereas groups sharing the same letter are not significantly different. Significance was determined by one-way ANOVA (** *p* < 0.01; *** *p* < 0.001).

To further optimize the four-phage cocktail for achieving sustained bacterial suppression with minimal bacterial regrowth, we monitored the cell density of MDR-UPEC isolates AT82 and AT84 during treatment with a range of phage doses (MOI 0.1, 1, 10, and 100). For each condition, the overall MOI of the cocktail was standardized across phage mixtures by proportionally adjusting the input of each component phage. Overall, the suppressive effects in both AT82 and AT84 was generally consistent with previous observations, although subtle dose-dependent variations were evident (Figure S3). In AT82, bacterial density declined rapidly at MOI 100 (Figure S3G; purple line) within the first 2–3 h; however, the density began to rise after approximately 9 h of incubation, indicating partial regrowth and possible survival of resistant subpopulations. At MOIs 1 and 10 (Figure S3G; orange and red lines), the onset of suppression was less pronounced than at MOI 100, but once established, the culture remained at low density for approximately 13 h before the density again began to increase. Interestingly, at the lowest dose (MOI 0.1; Figure S3G; yellow line), bacterial suppression initiated more slowly but persisted for the longest duration, maintaining reduced cell densities for up to 16 h. In AT84, cocktail activity was more uniform across doses. Similar to AT82, higher MOIs achieved rapid clearance during the early incubation, but by 16 h, all treatments converged to comparable inhibition levels, indicating consistent endpoint suppression regardless of initial phage input. AUC analysis supported these observations, showing significant growth reduction in all phage-treated conditions compared with the no-phage control (*p* < 0.05; Figure 5a,c).

To verify that the optical density changes reflected loss of viable cells, we quantified colony-forming units (CFU) at 16, 24, and 48 h post-infection (hpi). In both strains, all MOI conditions yielded a substantial ~3-log reduction in viable bacteria relative to untreated controls at 16 hpi, supporting the trends observed in cell-density measurements. Notably, in AT82, higher MOIs resulted in higher viable-cell count, whereas culture treated at lower MOIs maintained significantly reduced CFU values (Figure 5b; 16 hpi, and Figure S3G). In AT84, CFU reduction was comparable across all doses (Figure 5d; 16 hpi, and Figure S3H), consistent with its uniform endpoint inhibition. During prolonged incubation, an approximately 1-log increase in viable cell counts was consistently observed by 48 hpi in both AT82 and AT84, particularly at higher MOIs (Figure 5b,d; 48 hpi). However, the treatment at MOI 0.1 maintained the lowest viable cell count relative to the higher MOIs. Together, these results demonstrate that the durability of cocktail-mediated suppression is influenced by both the bacterial host background and phage dosing, providing practical insights for determining optimal dosing strategies for therapeutic applications.

### 2.8. Phage Cocktail Reduced UPEC Invasion with Alterations in Proinflammatory Cytokine Expressions During Human Bladder Epithelium Infection

The dose-dependent assays assessing bacterial suppression by the four-phage cocktail in liquid culture demonstrated that lower phage doses were able to sustain prolonged suppression with minimal bacterial revival. In particular, an MOI of 0.1 provided stable and sustained suppression of both MDR-UPEC isolates AT82 and AT84 without causing rapid regrowth during extended treatment (Figure 5 and Figure S3). Based on this outcome, we selected this optimal phage dose to evaluate its prophylactic potential against MDR-UPEC isolates AT82 and AT84, as well as the UPEC model strain UTI89, in a bladder cell model to better approximate physiological conditions and to avoid overwhelming host cells with excess phage particles.

**Figure 6:**
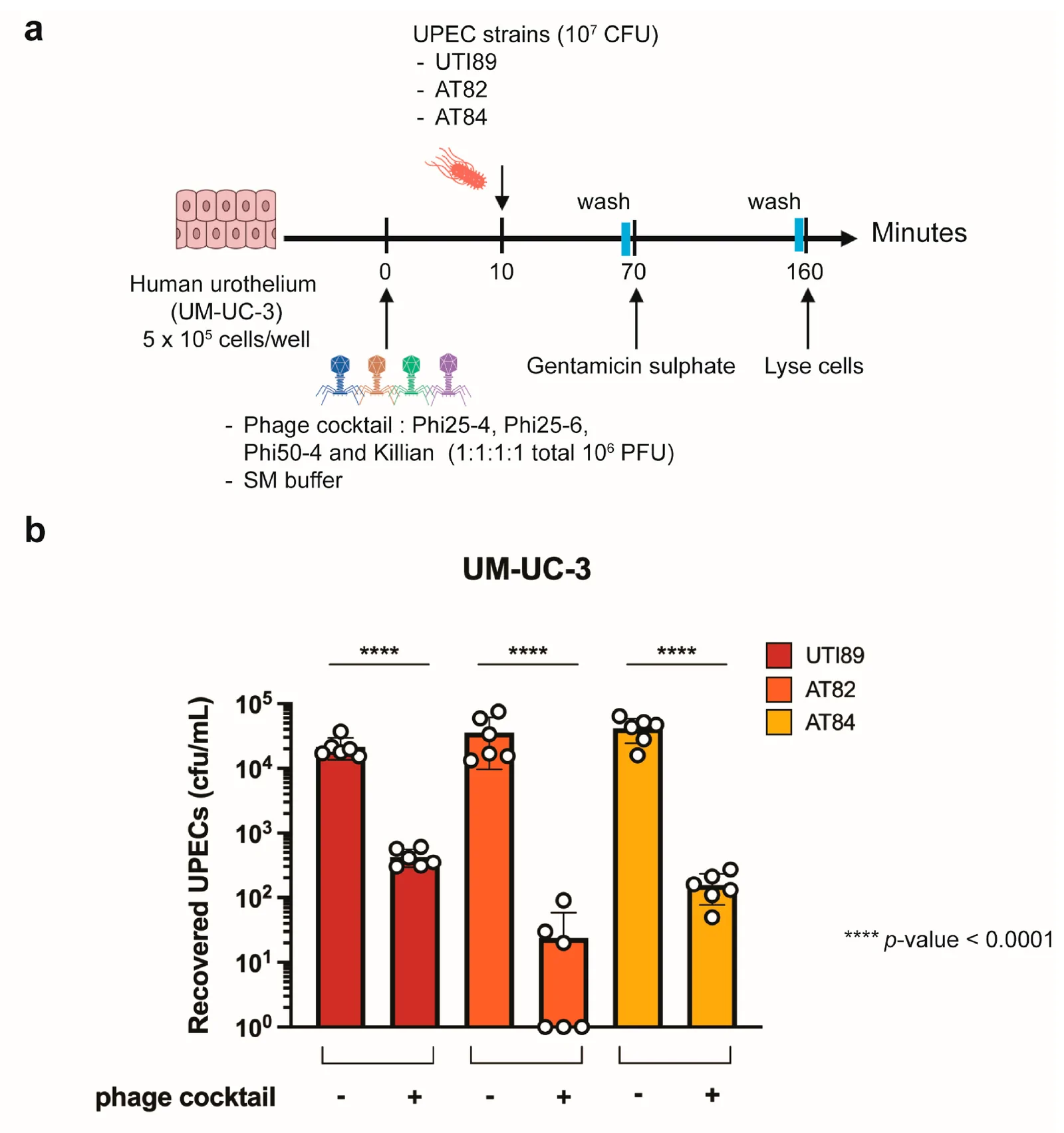
Phage cocktail reduces UPEC invasion into human bladder epithelial cells. (**a**) Schematic of the gentamicin protection invasion assay. UM−UC−3 bladder epithelial cells (5 × 10^5^ cells/well) were pretreated for 10 min with the four-phage cocktail (Phi25−4, Phi25−6, Phi50−4, and Killian; 1:1:1:1, total 10^6^ PFU), followed by infection with UPEC strains UTI89, AT82, or AT84 (10^7^ CFU). After 60 min of infection, extracellular bacteria were removed, and gentamicin was added for 90 min to kill non-internalized bacteria. Cells were then lysed to enumerate intracellular UPEC. (**b**) Recovered intracellular UPEC (log CFU/mL) from UM−UC−3 cells with or without phage pretreatment. Pretreatment with the phage cocktail significantly reduced invasion for all three UPEC strains compared with the untreated condition. Bars represent mean ± SD from three independent experiments. Statistical significance: **** *p* < 0.0001.

Since UPEC invasion into the host bladder epithelium is a critical step in UPEC pathogenesis [28], we next investigated whether the phage cocktail could reduce UPEC invasion into human bladder epithelial cells. A gentamicin protection assay was performed to determine the intracellular (invading) population of bacterial pathogens, including UPEC, as previously described [29]. Our data showed that prophylactic administration of the phage cocktail (MOI 0.1) into the human bladder cell line UM-UC-3 10 min prior to infection, significantly reduced intracellular UPEC in all strains tested, resulting in approximately 2–3 log reduction relative to untreated groups (Figure 6). Among the isolates, MDR-UPEC isolates AT82 is most susceptible to the prophylactic treatment, resulting in the largest decrease in recovered cells, while reductions in AT84 and UTI89 were slightly less pronounced. These findings suggest that prophylactic application of the phage cocktail rapidly eradicates extracellular UPEC populations in the cultures, thereby lowering subsequent bacterial invasion into human bladder epithelial cells.

**Figure 7:**
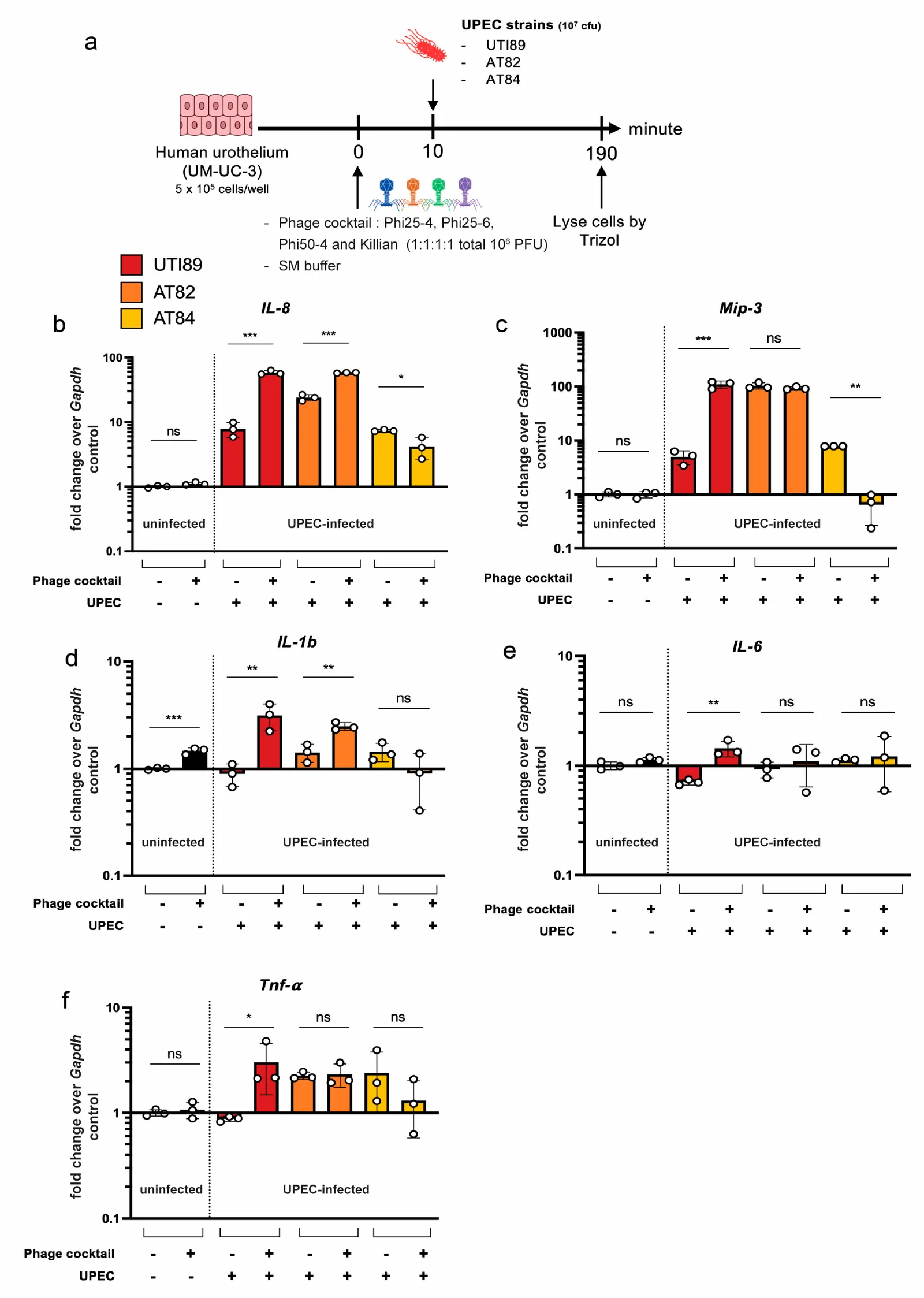
Effect of the phage cocktail on cytokine gene expression in human bladder epithelial cells during UPEC infection. (**a**) Schematic of cytokine-response experiment. UM−UC−3 cells (5 × 10^5^ cells/well) were pretreated for 10 min with the four-phage cocktail (Phi25−4, Phi25−6, Phi50−4, and Killian; 1:1:1:1, total 10^6^ PFU) before infection with UTI89, AT82, or AT84 (10^7^ CFU). After 180 min of infection, cells were lysed for RNA extraction and cytokine gene expression analysis. (**b**–**f**) Relative mRNA expression of IL−8 (**b**), MIP−3 (**c**), IL−1β (**d**), IL−6 (**e**), and TNF−α (**f**). In the absence of UPEC, phage cocktail treatment did not induce major cytokine changes. During UPEC infection, phage-pretreated cells exhibited altered cytokine responses in a strain-dependent manner, with pronounced induction in UTI89-infected and AT82-infected cells, while AT84-infected cells showed a more moderate response and reduced MIP−3 expression. Bars represent mean ± SD from three independent experiments. Statistical significance: ns, not significant; * *p* < 0.05; ** *p* < 0.01; *** *p* < 0.001.

Next, we assessed the immunomodulatory properties of the phage cocktail in human bladder epithelium in the presence and absence of UPEC infection. In uninfected UM-UC-3 cells, administration of the phage cocktail did not significantly alter the expression of key proinflammatory cytokine genes (*TNF-α*, *MIP-3*, *IL-6*, and *IL-8*), although a slight increase in *IL-1β* gene expression was observed. In contrast, variation in cytokine gene expression when UM-UC-3 cells were pretreated with the phage cocktail prior to UPEC infection can be detected (Figure 7). Expression of these cytokine genes (*TNF-α*, *MIP-3*, *IL-1β*, *IL-6*, and *IL-8*) significantly increased during infection; however, the extent of alteration varied among genes and across UPEC strains. In particular, *MIP-3* and *IL-8* expression was significantly downregulated in cells infected with AT84, suggesting strain-specific immunomodulatory responses to phage pretreatment.

## 3. Discussion

Urinary tract infections caused by UPEC remain among the most common bacterial infections worldwide and are increasingly complicated by the spread of MDR strains [30,31,32,33,34,35]. In the clinical setting, MDR-UPECs frequently exhibit resistance to several major antibiotic classes, including aminoglycosides, β-lactams, carbapenems and fluoroquinolones, thereby limiting treatment options [6,10,11,12,13,14,15]. In this study, among the clinical isolates collected from patients admitted at Maharaj Nakorn Chiang Mai Hospital (MNCMH), Chiang Mai, Thailand, three strains were identified as multidrug-resistant. These isolates encode β-lactamases, aminoglycoside-modifying enzymes, and quinolone resistance determinants, and notably some include resistance to colistin, a last-resort antibiotic. Such resistance profiles are consistent with UPEC strains currently encountered in hospital settings, in which treatment failures often arise due to resistance to third-generation cephalosporins and fluoroquinolones [10,11,12,13,14,15]. These limitations emphasize the urgent need for alternative therapeutic strategies that either act through mechanisms distinct from antibiotics or can complement existing therapies. In this study, we systematically customized a phage cocktail targeting these MDR-UPECs from our coliphage library and identified four potent lytic coliphages, Phi25-4, Phi25-6, Phi50-4 and Killian, that together effectively eradicated the MDR-UPEC isolates in both in vitro assays and a human bladder uroepithelial cell culture model.

Although phages exhibit high host specificity, which is a valuable therapeutic advantage, this same specificity can impose limitations on broad applicability [36,37]. In our systematic host-range screening of the phage library against a clinical UPEC panel, we noticed that although many isolates were susceptible to multiple phages, certain strains, including AT5 and MDR-AT72, were resistant to all phages tested. These findings highlight the substantial diversity within UPEC populations and suggest that even extensive phage collections may not cover all circulating clinical strains. These phage-resistant isolates therefore represent useful bacterial hosts for future phage discovery efforts aimed to expanding host-range coverage. Incorporating AT5 and MDR-AT72 as enrichment hosts during phage isolation may facilitate the targeted discovery of new phages capable of infecting these strains.

We previously proposed that combining genetically diverse phages in a cocktail would more effectively suppress bacterial regrowth and reduce the emergence of phage-resistant strains during prolonged exposure since simultaneously mutations conferring resistance to multiple phages are barely occurred [21]. Among the four selected phages, each exhibited strong lytic activity against the MDR-UPEC isolates individually, with Phi50-4 exhibiting the most favorable characteristics, including the fastest adsorption rate, the shortest latent period, and the largest burst size, suggesting the highest compatibility of phage-host interactions [38]. However, none of them alone prevented bacterial regrowth throughout an extended incubation. This emphasizes that single-phage therapy is insufficient for sustained suppression. Whole-genome sequencing and comparative genomic analyses revealed that all four phages were strictly lytic and belonged to two clearly distinct lineages. We found that genetic diversity among phage components contributed partially, but not uniformly, to cocktail performance. Certain pairings of phages from different lineages markedly enhanced the overall cocktail efficiency, such as Phi25-6 + Phi50-4 and Phi50-4 + Killian, but less likely in other cross-lineage combinations Phi25-4 + Phi25-6 and Phi25-4 + Killian. This pattern suggests possible virogenesis incompatibility that may obscure or diminish the replication efficiency of particular phage combinations [39,40,41,42,43]. Further investigation into phage-phage compatibility will be required to provide insights into rational cocktail design. Notably, the four-phage formulation provided the most robust and prolonged suppression, achieving >80% reduction in bacterial growth compared with untreated controls, with minimal emergence of phage-resistant isolates, and consistently outperforming smaller combinations. This enhanced potency likely arises not only from genetic divergence among the phages but also from complementary infection dynamics, including differences in adsorption frequencies and burst sizes that collectively maintain pressure on bacterial populations. Although the genome analyses confirmed the absence of virulence or lysogenic related-genes, further evaluation remain necessary to validate long-term safety. Future studies should include lysogeny and generalized transduction assays, as well as stability testing in simulated urinary conditions. In addition, since Killian previously showed synergy with antibiotics [24], it would also be valuable to determine whether such synergistic effects persist in the cocktail context.

Previous investigations have explored therapeutic agents, including phages, using human bladder cell infection models of UPEC UTI [29,44,45]. Current experimental and clinical evidence indicates that phage therapy is generally well tolerated and has the potential to effectively reduce bacterial burden. However, therapeutic outcomes remain variable, as several factors, including phage selection, cocktail composition, dose, and route of administration, can influence treatment efficacy [46,47,48]. Phage cocktails are particularly promising because combining phages with diverse biological and genetic characteristics can broaden antibacterial activity and potentially limit the emergence of phage-resistant populations. Accordingly, recent reviews have highlighted phage-based approaches as promising alternative or complementary strategies for treating UTIs, particularly those caused by antibiotic-resistant bacteria [46,47,48,49,50]. Furthermore, studies using genetically diverse lytic phages have demonstrated their ability to reduce UPEC invasion of human bladder epithelial cells and bacterial colonization in experimental UTI models [51]. Consistent with these previous observations, our findings demonstrate that the four-phage cocktail exhibited stronger and more sustained inhibition of MDR UPEC compared with individual and partial phage combinations, while also significantly reducing bacterial invasion of human bladder epithelial cells. However, given that diverse factors can influence the therapeutic outcomes of phage therapy, further investigation of the therapeutic efficacy of this cocktail under physiologically relevant conditions is required.

In this study, we prophylactically treated a monolayer of human bladder urothelium (UM-UC-3) with the phage cocktail and found that pretreatment with the phage cocktail significantly reduced UPEC invasion, lowering intracellular bacterial counts by 2–3 log10 compared with untreated cells. These findings suggest that phage presence at the bladder urothelial surface limits subsequent UPEC invasion, potentially reducing the establishment of intracellular bacterial reservoirs, a major contributor to recurrent UTIs. We also observed mild, strain-dependent alterations in proinflammatory cytokine mRNA expression in UPEC-infected UM-UC-3 cells exposed to phages. For example, *MIP3* expression decreased during AT84 infection, supporting previous observations from our group that pathogen-phage interactions might influence eukaryotic cellular signaling in a strain-dependent manner [22]. Importantly, in the absence of UPEC infection, the phage cocktail alone did not induce proinflammatory cytokines, except for a mild increase in *IL1b*, indicating a favorable safety of this phage cocktail when encountering the human bladder epithelium. Nonetheless, a key limitation of the current model is that it relies only on one cell type (a monolayer of human bladder urothelial cells), which does not capture the cellular heterogeneity or architectural complexity of the human bladder. The development and application of human bladder organoid systems may therefore provide more physiologically relevant platforms for future investigations of phage therapy in UTI [52]. Overall, our findings support the current evidence that rationally selected and genetically diverse phage cocktails may provide a useful strategy for controlling MDR UPEC-associated UTIs, although further in vivo and clinical studies are required to determine the most effective treatment conditions.

## 4. Materials and Methods

### 4.1. Bacterial Strains and Growth Conditions

Clinical UPEC strains were isolated from urine cultures of patients diagnosed with UTIs as shown in Supplementary Table S1. Briefly, bacterial isolates were initially recovered from the samples and differentiated on Macconkey agar, followed by the detection of UPEC-associated signature genes and further characterized for the presence of UPEC virulence-associated genes [29]. The whole genome sequences of two isolates (AT82 and AT84) were reported [23]. All UPEC strains were cultured in Luria–Bertani (LB) broth (10 g/L tryptone, 5 g/L yeast extract, and 10 g/L NaCl; Difco, United States for 16–18 h at 37 °C with shaking. A total of 17 clinical UPEC isolates in this study were obtained from urine culture of patients admitted to Maharaj Nakorn Chiang Mai Hospital (MNCMH), Thailand in 2020 (Approval No. 097/2567 and 253/2568 by the Research Ethics Committee, Faculty of Medicine, Chiang Mai University).

### 4.2. Phage Isolation and Purification

Wastewater samples were used as the source of phages for the isolation step. One milliliter of samples was first filtered through a 0.45 µm filter and mixed with 5 mL of an overnight culture of UPEC UTI89, then incubated at 37 °C overnight to enrich phages. The enrichment cultures were centrifuged at 8000 rpm for 20 min and filtered to remove bacterial cells, and the resulting phage-containing supernatant was subjected to a 25–100% Percoll gradient and centrifuged at 100,000× *g* for 2 h. Fractions from the gradient were used to infect host cells and were plated using the double-layer agar method to isolate individual phages. The double-layer agar method was performed by mixing 10 µL of phage, 100 µL of UPEC, and 5 mL of 0.35% LB agar, then pouring the mixture onto an LB plate. After incubation at 37 °C overnight, a single, well-defined, translucent plaque was picked and purified through at least three successive rounds using the double-layer agar method, or until the plaque morphology was homogeneous.

### 4.3. Minimum Inhibitory Concentration (MIC) Assay

The antibiotic susceptibility of UPEC isolates (AT1–6, AT71–74, and AT79–86) was determined using a broth microdilution assay in 96-well microtiter plates. Two-fold serial dilutions of six antibiotics, including amikacin, ceftriaxone, ciprofloxacin, colistin, gentamicin, and meropenem, were prepared in LB medium. Overnight bacterial cultures were diluted to an OD_600_ of 0.2 and subsequently diluted 1:10 in LB medium. One microliter of the diluted bacterial suspension was added to each well containing the antibiotic dilutions. Wells containing bacteria without antibiotics served as growth controls, while LB medium without bacteria served as sterility controls. The plates were incubated at 37 °C for 24 h, and the minimum inhibitory concentration (MIC) was defined as the lowest antibiotic concentration at which no visible bacterial growth was observed compared with the growth control. All assays were performed in triplicate, and resistance was interpreted according to CLSI breakpoint criteria.

### 4.4. Phage Host Range Determination

The phage host range was determined using a spot test against the same UPEC panel by spotting 5 µL of each phage lysate (10^8^ pfu/mL) onto lawns of the UPEC isolates prepared using the double-layer agar method. After overnight incubation at 37 °C, lysis at the spot site was recorded. Clear and complete lysis was classified as strong infectivity (++), turbid or incomplete lysis was classified as intermediate infectivity (+), and the absence of visible lysis was classified as no detectable infectivity. Phage isolates were designated Phi25-1 to Phi25-6, Phi50-1 to Phi50-5, Phi75-1 to Phi75-5, Phi100-1 to Phi100-5, and Killian. The leading number in each designation corresponds to the Percoll gradient fraction (25–100%) from which the phage was recovered. The experiments were carried out in triplicate.

### 4.5. Phage Clustering Based on Host Similarity

To identify groups of phages with related host specificities, we first assigned numerical scores to the infectivity profile of each phage, which were organized into a binary matrix, where infection was recorded as 1 and no infection as 0 for every host strain tested. These scores were then used to construct a host specificity matrix, which we analyzed using hierarchical clustering with the Jaccard distance metric in Morpheus https://software.broadinstitute.org/morpheus (accessed on 12 June 2025).

### 4.6. Identification of Phages with Strong Lytic Activity

To assess phage lytic efficacy, killing assays were conducted in 96-well plates using UPEC strains AT82 and AT84. The log phase of UPEC (90 µL) was co-cultured with phage (90 µL) and 10X LB (20 µL) at an MOI of 1 in 96-well plates, and bacterial growth was monitored by measuring OD_600_ at 10-min intervals, 37 °C for 16 h. Killing efficiency was quantified by calculating the area under the curve (AUC) using GraphPad Prism 10. The AUC values were compared using a one-way ANOVA, and *p*-values were corrected for multiple comparisons. Phages that reduced AUC by at least 50% relative to control (No phage) were considered effective candidates for inclusion in cocktail formulations. All assays were performed in triplicate.

### 4.7. Biological Properties of Phage Candidates

For Transmission electron microscopy (TEM), phage lysates were precipitated for 16 h with 1 M NaCl and 10% (*w*/*v*) polyethylene glycol and then centrifuged at 9000 rpm for 20 min. Pellets were resuspended in SM buffer. Phage particles were adsorbed onto carbon-coated copper grids, negatively stained with 2% (*w*/*v*) uranyl acetate, and visualized by transmission electron microscopy (TEM; Hitachi HT7700, Hitachi High-Tech Corporation, Tokyo, Japan).

Phage adsorption kinetics were determined by mixing log-phase MDR-UPEC AT82 (OD_600_ ≈ 0.4) with phage at MOI 0.01 in LB broth at 37 °C. At 5-min intervals over 50 min, 200 µL samples were withdrawn and filtered through 0.45 µm membranes to remove adsorbed phages and host cells. The sample from each time point was assayed by the double-layer agar method to determine the number of unadsorbed phages.

Latent period and burst size were determined by the one-step growth experiment. The MDR-UPEC AT82 (OD_600_ = 0.4) was infected with phage at MOI 0.01, followed by centrifugation at 9000 rpm for 2 min to remove free phages. Supernatants were used in the double-layer agar method to quantify unadsorbed phage, while the pellet was washed once in LB, resuspended in 20 mL fresh LB, and incubated at 37 °C. Aliquots (200 µL) were collected every 5 min, filtered (0.45 µm), and phage titers were determined by spot titer assay. The latent period was defined as the interval from initial adsorption until the first detectable rise in extracellular phage concentration. Burst size was calculated using the formula:

$$Burst\;size=\;\frac{A-B}{Infected\;cell}$$

where *A* is the average phage particle at plateau after the burst, *B* is the average phage particle at plateau before the burst, and the number of infected cells was estimated as the number of phages added minus the number of unadsorbed phages, and expressed in units of PFU/infected cell [22].

For phage DNA extraction, phage lysates were precipitated with 10% (*w*/*v*) polyethylene glycol and 1 M NaCl and pelleted by centrifugation (8500 rpm, 10 min). Pellets were resuspended in SM buffer and treated with DNase I (10 U) and RNase A (0.1 mg mL^−1^). Nuclease activity was quenched by adding EDTA to a final concentration of 20 mM, after which capsids were disrupted with proteinase K (0.5 mg mL^−1^) and SDS (0.5%) at 60 °C for 1 h. Genomic DNA was extracted twice with an equal volume of phenol–chloroform–isoamyl alcohol (25:24:1) with gentle inversion, followed by centrifugation (15,000× *g*, 20 min) and collection of the aqueous phase. DNA was precipitated with 1/10 volume of 3 M sodium acetate and two volumes of cold absolute ethanol at −20 °C for 3 h, pelleted (18,000× *g*, 20 min), rinsed with 70% ethanol, centrifuged again (18,000× *g*, 20 min), air-dried, and dissolved in TE buffer.

### 4.8. Bacterial Whole-Genome Sequencing and Bioinformatic Analysis of Clinical UPEC Isolates AT82 and AT84

Bacterial DNA extraction and whole-genome sequencing were performed as described by Buddhasiri S [23]. Briefly, genomic DNA was extracted and purified using a spin-column DNA extraction kit. Whole-genome sequencing was conducted on the Illumina NovaSeq platform. Raw reads were quality-checked, trimmed to remove adapters and low-quality bases, and subsequently assembled into draft genomes. Acquired antimicrobial resistance genes were identified using ResFinder v4.7.2 with thresholds of ≥90% sequence identity and ≥60% minimum length coverage, while *E. coli* point mutations associated with resistance were analyzed using PointFinder v4.1.1 [53,54].

### 4.9. Whole-Genome Sequencing and Bioinformatic Analysis of Phage Candidates

Phage genomic DNA was extracted from high-titer lysates (around 10^9^ PFU/mL). Before extraction, the lysates were treated with DNase I and RNase A to remove any remaining host nucleic acids, then filtered through 0.45 µm filters. DNA was purified using the phenol–chloroform extraction method followed by ethanol precipitation. The DNA quality and quantity were checked using a Nanodrop spectrophotometer to make sure the samples were clean and concentrated enough for sequencing. Sequencing was performed on the Illumina platform with paired-end reads. The reads were assembled de novo using Unicycler (v. 0.0.5), which works well for circular or near-circular genomes such as phages. The quality of the assemblies was assessed using QUAST (v. 5.2.0), and the reads were mapped back to the assemblies to confirm coverage and accuracy. The annotated genomes were generated using Bakta (v. 1.8.1), which automatically predicts coding sequences, tRNAs, and other relevant features based on curated databases. Toxin and virulence genes were predicted using CSM-Toxin v1.0.1, VFanalyzer v4.0, and VirulenceFinder v2.0. Antimicrobial resistance genes were identified using ResFinder version 4.7.2. The final circular genome maps were visualized using Proksee [55], allowing for easy inspection of genome structure, GC content, and predicted functional modules. Comparative analysis between the isolated phages was done using DiGAlign [56] to visualize genome alignment and overall gene organization. Pairwise nucleotide similarity was calculated using VIRIDIC [27] to estimate intergenomic identity. All annotated genome sequences have been submitted to GenBank under accession numbers as follows: PX393109 (Phi25-4), PX393108 (Phi25-6), PX393110 (Phi50-4), and OQ446694 (Killian).

### 4.10. Phage Cocktail Formulation

Phage cocktails were formulated to evaluate the combined lytic activity of multiple phages against MDR *E. coli*. Four candidate phages (Phi25-4, Phi25-6, Phi50-4, and Killian) were tested by the Killing assay in all possible two-, three-, and four-member combinations. For each formulation, phages were mixed in equal proportions and adjusted to yield a total multiplicity of infection (MOI) of 1. UPEC strains AT82 and AT84 (OD_600_ ≈ 0.4) were inoculated into 96-well plates and challenged with each cocktail. Bacterial growth was monitored at 37 °C incubation, and OD_600_ values were recorded at 10-min intervals for 16 h using a microplate reader. Growth inhibition was quantified by calculating the area under the curve (AUC) for each replicate. Statistical analysis was performed by one-way ANOVA, and the most effective cocktail was defined as the combination achieving the greatest and statistically significant reduction in bacterial growth compared to the no-phage control.

### 4.11. Dose-Dependent Killing by the Phage Cocktail

The bactericidal activity of the optimized phage cocktail was further evaluated across a range of MOIs. AT82 and AT84 cultures (OD_600_ ≈ 0.4) were infected with the four-phage cocktail at MOIs of 0.01, 0.1, 1, 10, and 100. Bacterial growth was monitored in 96-well plates under the same conditions as above, and OD_600_ values were collected over 16 h to generate killing curves. Growth inhibition was quantified by AUC analysis, and dose dependence was assessed by comparing AUC values across MOIs relative to the no-phage control. To confirm bacterial viability following phage treatment, samples were collected at 16, 24, and 48 h post-infection. Each sample was serially diluted tenfold in sterile normal saline. A 10 µL aliquot of each dilution was spotted onto LB agar plates and allowed to dry before incubation overnight at 37 °C. Colonies were counted from spots containing a countable number of colonies, and bacterial viability was expressed as colony-forming units per milliliter (CFU/mL). Data were expressed as mean ± SD from independent biological triplicates.

### 4.12. Gentamicin Protection Assay for UPEC Invasion

The complete growth medium contained Roswell Park Memorial Institute 1640 (RPMI 1640; 2.05 mM L-glutamine, Cytiva, Logan, UT, USA), 10% fetal bovine serum (FBS; Cytiva, Pasching, Austria), and 1% penicillin/streptomycin (Cytiva, Pasching, Austria), was used to grow the human bladder epithelium UM-UC-3 (ATCC CRL-1749) in a T75 flask at 37 °C with 5% CO_2_. Then, the cells were split into a 24-well plate (10^5^ cells per well) and incubated for 24 h. Cell culture media were replaced with RPMI-1640 without FBS and antibiotics before the gentamicin protection invasion assay. 10 μL of high-titer phage solution (10^8^ PFU/mL) was added to each well (10^6^ PFU/well), and the plate was incubated at 37 °C for 10 min. The phage-pretreated UM-UC-3 cells were infected with 100 μL of UPEC strains (10^8^ CFU/mL; 10^7^ CFU/well), including UTI89, AT82, and AT84, corresponding to a total MOI of 0.1 based on the phage-to-UPEC ratio, and incubated at 37 °C for 1 h. Each well was washed twice with sterile phosphate-buffered saline (PBS) before adding 0.5 mL of media containing 100 μg/mL gentamicin sulfate (AppliChem, Darmstadt, Germany), and incubated at 37 °C for 90 min. After washing with PBS, 0.5 mL of 1% Triton-X-100 (Thermo Fisher Scientific, Waltham, MA, USA) was used to lyse the infected UM-UC-3 cells. The recovered CFU/mL of UPEC strains were enumerated with a standard serial dilution technique on the LB agar.

### 4.13. Proinflammatory Gene Expression by a qPCR

UM-UC-3 cells were cultivated in the complete growth medium as described above to reach a density of approximately 10^6^ cells per well in 6-well plates. The plates were incubated at 37 °C with 5% CO_2_ for 24 h as previously described [29]. The media were replaced with FBS and antibiotic-free complete growth medium. Then, 10 μL of high-titer phage solution (10^8^ PFU/mL) was added to each well (10^6^ PFU/well), and the plate was incubated at 37 °C for 10 min. The phage-pretreated UM-UC-3 cells were infected by The phage-pretreated UM-UC-3 cells were infected with 100 μL of UPEC strains (10^8^ CFU/mL), including UTI89, AT82, and AT84 corresponding to a total MOI of 0.1 based on the phage-to-bacterium ratio, and incubated at 37 °C for 3 h. Cells were lysed with 1 mL of TRIzol reagent (Thermo Fisher Scientific, USA) to harvest RNA according to the manufacturer’s instructions. The RevertAid First Strand cDNA kit (Thermo Fisher Scientific, Vilnius, Lithuania) was used to synthesize cDNA. Then, qPCR was performed using SYBR Green-based real-time PCR (Bioline, Memphis, Tennessee, USA) on the ViiA7 Real-Time PCR machine (Applied Biosystems, United States). The relative fold change in mRNA expression of proinflammatory cytokine genes (*IL8*, *MIP3*, *IL1β*, *IL6*, and *TNF-α*) was determined using the comparative Ct method (the 2^−ΔΔCT^ by using the *GAPDH* as the housekeeping gene) as previously described [29]. The primers are presented in the Supplementary Table S3.

## 5. Conclusions

Although numerous *E. coli* phages have been studied for decades, relatively few have been isolated and developed specifically to target UPEC strains. Compared with laboratory strains, UPEC strains exhibit distinct virulence traits and resistance patterns, emphasizing the importance of isolating phages directly against clinical isolates. In response to this challenge, we have previously established a pipeline for rapid formulation of a genetically diverse phage cocktail against a UPEC-UTI model. Moreover, among phages in our phage library, we characterized the phage Killian and demonstrated its synergistic interaction with certain antibiotics. Consequently, expanding the phage library to broaden host coverage and enabling rational phage cocktail design is highly necessary. Here, we systematically customized a four-phage cocktail from our coliphage library specifically targeting MDR-UPEC isolates from hospitalized patients. The cocktail comprises virulent phages Phi25-4, Phi25-6, Phi50-4, and Killian, which cluster into two distinct viral lineages. Individually, these phages display subtle variations in lysis kinetics and are insufficient to sustain bacterial suppression during prolonged treatment. In contrast, when all four phages are combined, the resulting cocktail achieves durable bacterial suppression with minimal surviving cells over a 48-h period and prophylactically prevents UPEC invasion in human bladder epithelial cells. The cocktail itself elicits minimal effect on pro-inflammatory gene expression, suggesting its safety for mammalian cells.

## Figures and Tables

**Table 1:** Antibiotic resistance genes detected in *E. coli* AT82 and AT84.

Resistance Mechanism Gene	AMR Gene Family	Target Drug Class	Number of Gene	
			**AT82**	**AT84**
**Aminoglycoside modifying**				
*aac(3)-IId*	aminoglycoside antibiotic	Aminoglycoside	1	1
*aph(3″)-Ib (strA)*	aminoglycoside antibiotic	Aminoglycoside	1	1
*aph(6)-Id (strB)*	aminoglycoside antibiotic	Aminoglycoside	1	1
**Multidrug efflux complex**				
*acrAB-TolC* efflux	RND efflux system	MDR	3	3
*acrEF-TolC* efflux	RND efflux system	MDR	4	4
*cpxR/cpxA* system	RND efflux system	MDR	2	2
*emrAB-TolC*	MFS efflux pump	MDR	2	2
*emrD*	MFS efflux pump	Phenicol	0	1
*emrE*	SMR efflux pump	Macrolide	2	2
*emrKY-TolC*	MFS efflux pump	Tetracycline	2	2
*evgSA* system	RND/MFS efflux pump	MDR	2	2
*marRAB*	RND efflux pump	MDR	2	1
*mdfA*	MFS efflux pump	MDR	1	1
**Multidrug transporter**				
*mdtABC*	RND efflux pump	Aminocoumarin (Novobiocin)	3	3
*mdtEF-TolC*	RND efflux pump	MDR	2	2
*mdtG*	MFS efflux pump	phosphonic acid (Fosfomycin)	1	1
*mdtH*	MFS efflux pump	fluoroquinolone (norfloxacin)	1	1
*mdtI, mdtJ*	SMR efflux pump	MDR	2	2
*mdtK*	MATE transporter	Fluoroquinolone	1	1
*mdtM*	MFS efflux pump	MDR	1	1
*mdtNOP*	MFS efflux pump	MDR	3	3
*msbA*	ABC efflux pump	Nitroimidazole antibiotic	1	1
*yojI*	ABC efflux pump	Peptide antibiotic	1	1
*acrS, acrD, baeR, baeS, emrR, robA, soxS*	Gene modulating antibiotic efflux	MDR	7	7
**Macrolide phosphotransferase**				
*ampH*	macrolide phosphotransferase	MDR	1	1
*mphB*	macrolide phosphotransferase	Macrolide antibiotic	1	1
**Quinolone**				
*qnrS1*	quinolone resistance	Fluoroquinolone antibiotic	1	1
**Sulfonamide**				
*sul2*	sulfonamide resistant	Sulfonamide antibiotic	1	1
**Tetracycline**				
*tetA, tetR*	MFS efflux pump	tetracycline antibiotic	2	2
**Trimethoprim**				
*dfrA14*	trimethoprim resistant dihydrofolate reductase	Diaminopyrimidine antibiotic	1	1
**β-lactamase**				
*ampC*	ampC-type beta-lactamase	Cephalosporin, Penicillin	1	1
*CTX-M-55*	CTX-M beta-lactamase	Cephalosporin	1	1
*EC-8*	EC beta-lactamase	Ccephalosporin	1	1
*TEM-1*	TEM beta-lactamase	Penicillin, Cephalosporin, Monobactam	1	1
*TEM-105*	TEM beta-lactamase	Penicillin, Cephalosporin, Monobactam	1	1
*PBP2*	Penicillin-binding protein	Penicillin	1	1
**Plasmid**				
IncQ1	IncQ-type plasmids	MDR	1	1
IncFIB	IncFIB plasmids carrying the resistance gene	MDR	1	1
IncFIC(FII)	IncFII-type multidrug resistant plasmids	MDR	1	1

**Table 2:** WGS-predicted resistant phenotype of UPEC strains AT82 and AT84.

Antimicrobial	Class	Genetic Background
Gentamicin	aminoglycoside	*aac(3)-IId*
Tobramycin	aminoglycoside	*aac(3)-IId*
Streptomycin	aminoglycoside	*aph(6)-Id, aph(3″)-Ib*
Dibekacin	aminoglycoside	*aac(3)-IId*
Netilmicin	aminoglycoside	*aac(3)-IId*
Apramycin	aminoglycoside	*aac(3)-IId*
Sisomicin	aminoglycoside	*aac(3)-IId*
Amoxicillin	beta-lactam	*blaCTX-M-55, blaTEM-1B*
Ampicillin	beta-lactam	*blaCTX-M-55, blaTEM-1B*
Cefepime	beta-lactam	*blaCTX-M-55*
Cefotaxime	beta-lactam	*blaCTX-M-55*
Ceftazidime	beta-lactam	*blaCTX-M-55*
Piperacillin	beta-lactam	*blaCTX-M-55, blaTEM-1B*
Aztreonam	beta-lactam	*blaCTX-M-55*
Ticarcillin	beta-lactam	*blaCTX-M-55, blaTEM-1B*
Ceftriaxone	beta-lactam	*blaCTX-M-55*
Cephalothin	beta-lactam	*blaTEM-1B*
Sulfamethoxazole	folate pathway antagonist	*sul2*
Trimethoprim	folate pathway antagonist	*dfrA14*
Hydrogen peroxide	peroxide	*sitABCD*
Ciprofloxacin	quinolone	*qnrS1*
Tetracycline	tetracycline	*tet(A)*
Doxycycline	tetracycline	*tet(A)*

## Data Availability

The original data presented in this study are included in the article and Supplementary Materials. The genome sequences generated in this study are publicly available in GenBank under the accession numbers: PX393109 (Phi25-4), PX393108 (Phi25-6), and PX393110 (Phi 50-4). Further inquiries can be directed to the corresponding author.

## Supplementary Materials

The following supporting information can be downloaded at: https://www.mdpi.com/article/10.3390/antibiotics15090870/s1, Figure S1: Host range profiles of UPEC-targeting phages against clinical and laboratory *E. coli* strains. Figure S2: Genomes and comparative genomics. Figure S3: Growth suppression profiles of MDR-UPEC treated with different phage combinations and multiplicities of infection. Table S1: Bacterial strains used in this study. Table S2: Minimum inhibitory concentrations (MICs) of antibiotics against clinically isolated UPEC. Table S3: List of qPCR primers used in this research.
