# Supplementary material for: Rational Design of a Phage Cocktail for Effective Control of Multidrug-Resistant Uropathogenic Escherichia coli from Hospitalized Patients
Source: Antibiotics (Basel). 2026 Sep 6;15(9):870. doi: 10.3390/antibiotics15090870 (PMC13603250; doi:10.3390/antibiotics15090870)
Supplement: Supplementary file 1 [file antibiotics-15-00870-s001.zip › antibiotics-4496298-supplementary.pdf]

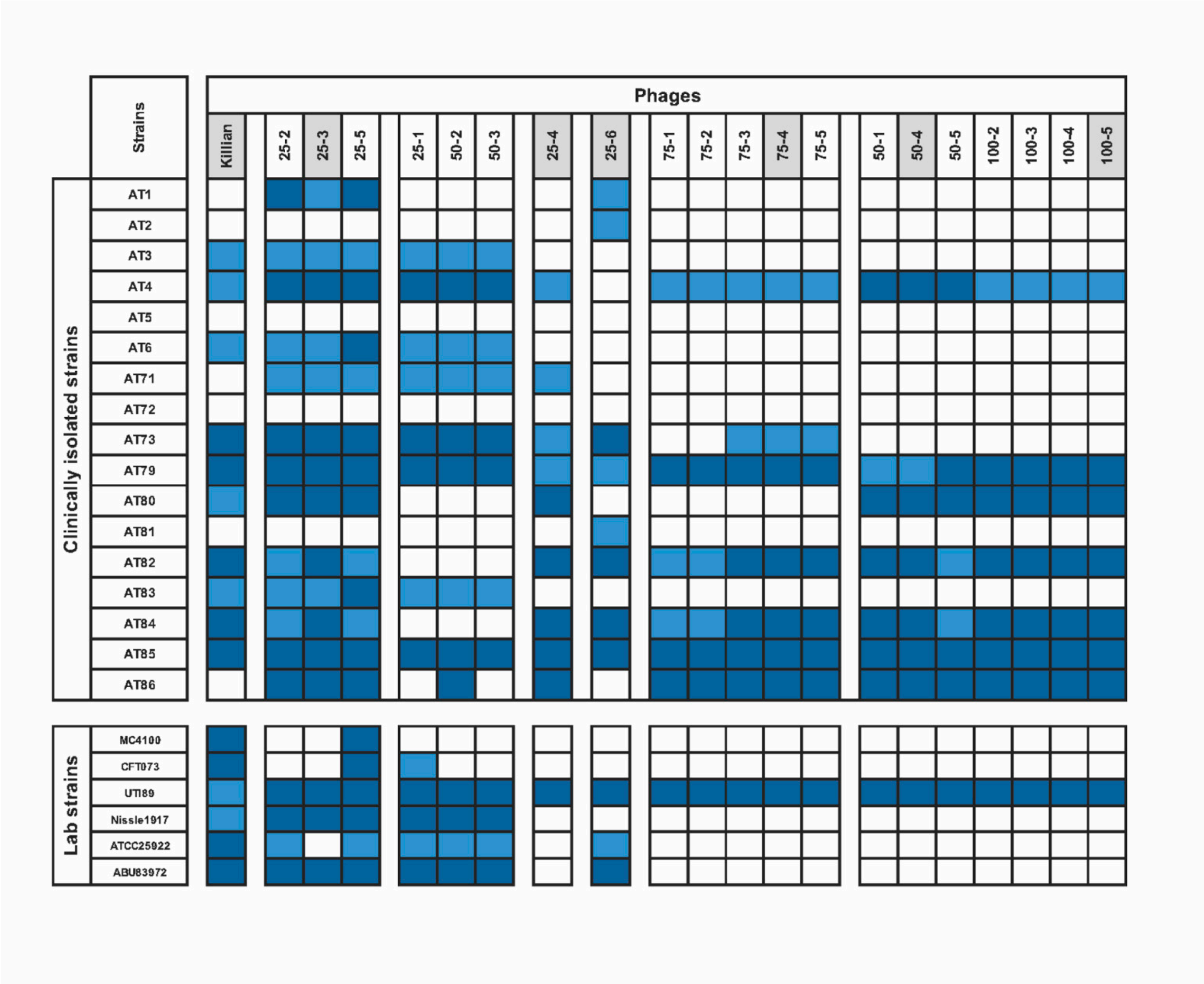

Figure S1. Host range profiles of UPEC-targeting phages against clinical and laboratory *E. coli* strains. Heatmap showing the infection outcomes of 20 isolated phages and Killian against a panel of 17 clinically isolated UPEC strains and 6 laboratory *E. coli* strains (MC4100, CFT073, UTI89, Nissle1917, ATCC25922, and ABU83972). Infection phenotypes were determined by spot testing on double-layer agar, and outcomes were scored as strong clearing (Dark blue), intermediate clearing (Light blue), or no infection (White). Rows represent bacterial strains and columns represent phages. The matrix highlights distinct infectivity patterns across isolates, reflecting strain-specific susceptibility and the overall diversity of the phage collection.

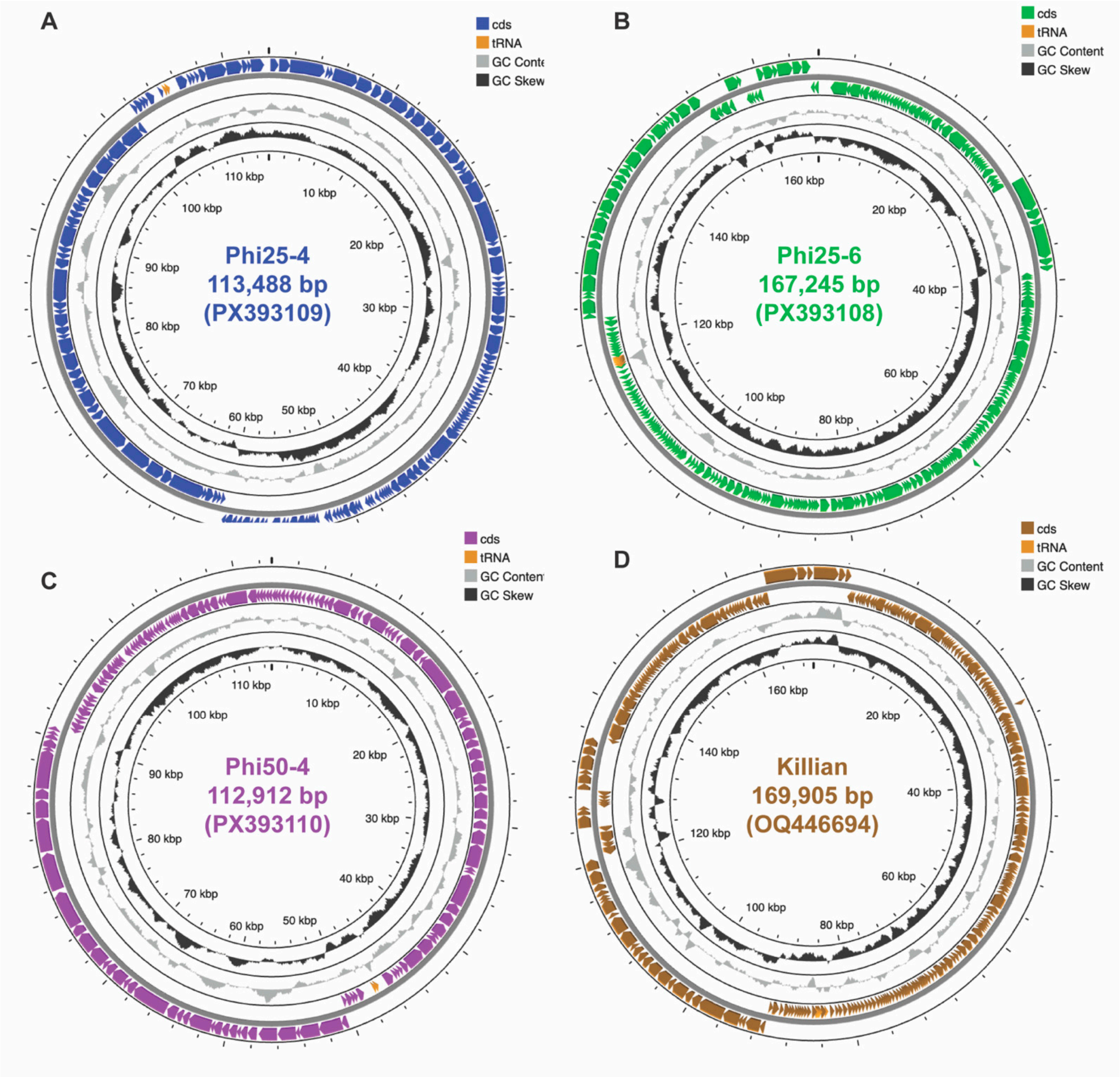

A
cds
tRNA
GC Conte
GC Skew
Phi25-4
113,488 bp
(PX393109)
B
cds
tRNA
GC Content
GC Skew
Phi25-6
167,245 bp
(PX393108)
C
cds
tRNA
GC Conten
GC Skew
Phi50-4
112,912 bp
(PX393110)
D
cds
tRNA
GC Content
GC Skew
Killian
169,905 bp
(OQ446694)

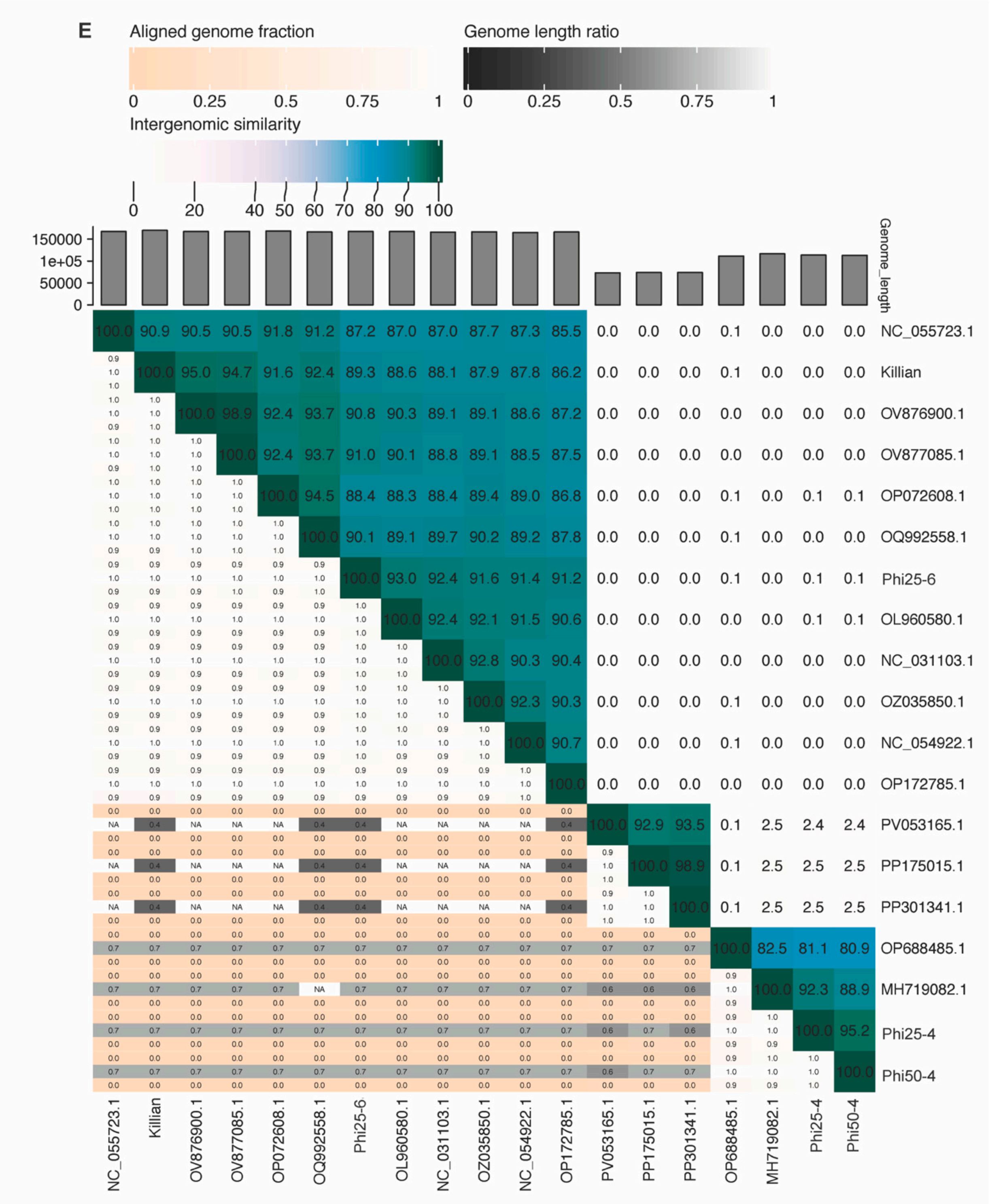

Figure S2. Genomes and comparative genomics. Circular genome maps of (A) *Escherichia* phage Phi25-4, (B) Phi25-6, (C) Phi50-4, and (D) Killian are shown with annotated coding sequences (CDSs), tRNA genes, GC content, and GC skew. The total genome sizes are indicated in the center of each map. (E) VIRIDIC heatmap showing pairwise intergenomic similarity and aligned genome fraction among isolated

phages and closely related reference phages. Phi25-4 clusters with Phi50-4, and Phi25-6 clusters with Killian, confirming two distinct phage lineages

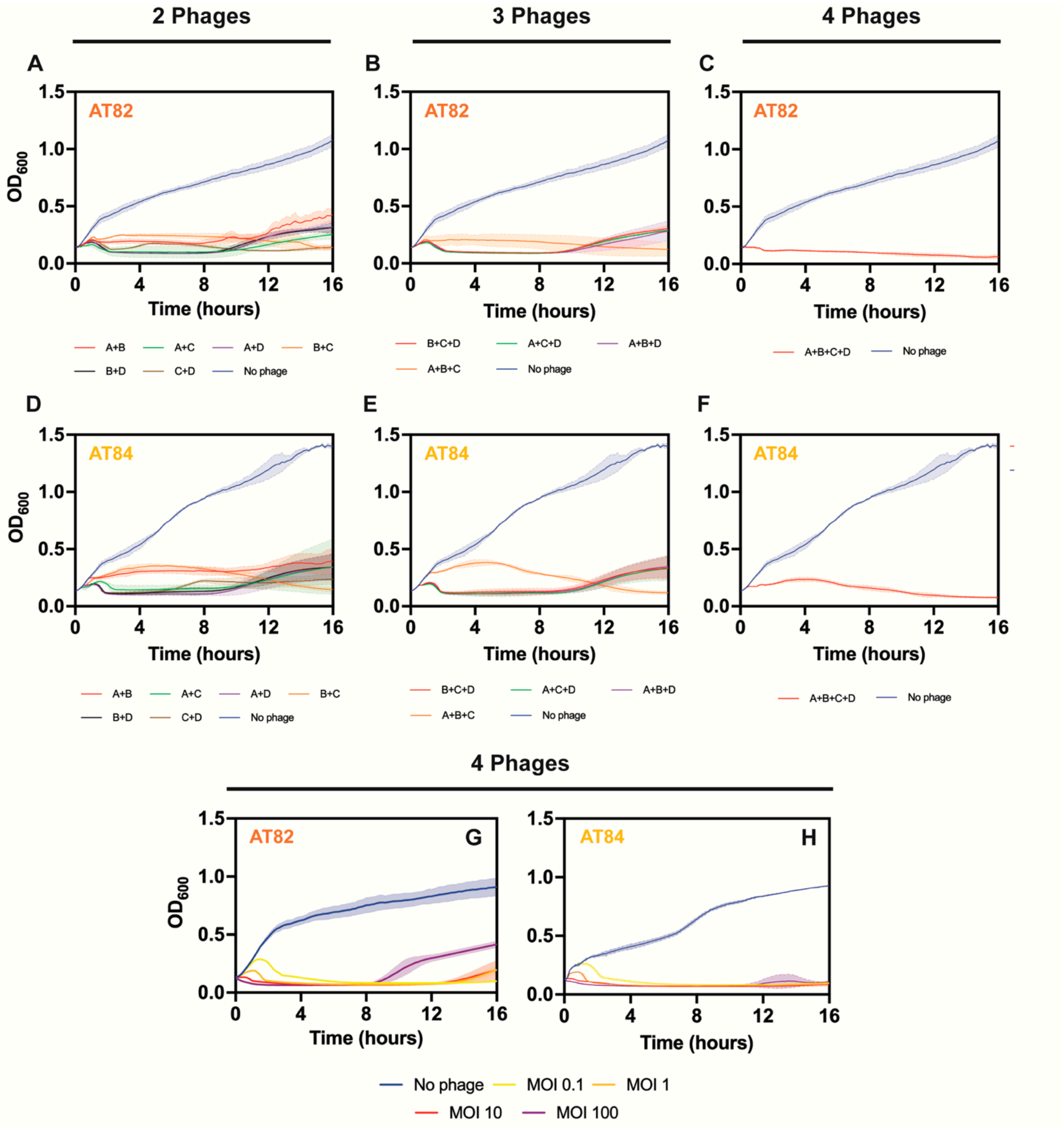

Figure S3. Growth suppression profiles of MDR-UPEC treated with different phage combinations and multiplicities of infection. (A–C) Killing curves of AT82 over 16 h following treatment with two-phage (A), three-phage (B), and four-phage (C) mixtures composed of phi25-4 (A), phi25-6 (B), phi50-4 (C), and

Killian (D) at a total MOI of 1. Each curve represents the mean from three biological replicates, with shaded areas denoting SD. The four-phage cocktail (A+B+C+D) maintained the most stable suppression across the monitoring period. **(D–F)** Killing curves of AT84 under the same treatment conditions, showing reduced regrowth as the number of phages in the mixture increased. Similar to AT82, the four-phage cocktail (A+B+C+D) produced the strongest and most durable inhibition. **(G–H)** Dose-dependent suppression of AT82 (G) and AT84 (H) by the four-phage cocktail at MOIs 0.1, 1, 10, and 100, compared with the no-phage control. Shaded areas represent SD from three independent experiments.

Table S1. Bacterial strains used in this study. All clinical UPEC strains used in this study were originally isolated from urine cultures of patients diagnosed with UTIs. Bacterial isolates were initially recovered and differentiated on Macconkey agar, followed by the detection of UPEC-associated signature genes (c3509, c3686, chuA, and uidA) and further characterized for the presence of UPEC virulence-associated genes (fimH, sfa, and iroN). (Tantibhadrasapa et. al., 2024)

Designation	Species	Relevant characteristic	Reference
AT1	*Escherichia coli*	MDR clinical isolate from urine culture a 67-year-old woman with urinary tract infection	This study
AT2	*Escherichia coli*	MDR clinical isolate from urine culture a 81-year-old man with urinary tract infection	This study
AT3	*Escherichia coli*	MDR clinical isolate from urine culture a 67-year-old man with urinary tract infection	This study
AT4	*Escherichia coli*	MDR clinical isolate from urine culture a 41-year-old woman with urinary tract infection	This study
AT5	*Escherichia coli*	MDR clinical isolate from urine culture a 74-year-old woman with urinary tract infection	This study
AT6	*Escherichia coli*	MDR clinical isolate from urine culture a 74-year-old woman with urinary tract infection	This study
AT71	*Escherichia coli*	MDR clinical isolate from urine culture a 90-year-old man with urinary tract infection	This study
AT72	*Escherichia coli*	MDR clinical isolate from urine culture a 63-year-old woman with urinary tract infection	This study
AT73	*Escherichia coli*	MDR clinical isolate from urine culture a 2-year-old boy with urinary tract infection	This study
AT74	*Escherichia coli*	MDR clinical isolate from urine culture a 86-year-old woman with urinary tract infection	This study
AT79	*Escherichia coli*	MDR clinical isolate from urine culture a 76-year-old woman with urinary tract infection	This study
AT80	*Escherichia coli*	MDR clinical isolate from urine culture a 91-year-old woman with urinary tract infection	This study
AT81	*Escherichia coli*	MDR clinical isolate from urine culture a 60-year-old man with urinary tract infection	This study
AT82	*Escherichia coli*	MDR clinical isolate from urine culture a 83-year-old man with urinary tract infection	PMID: 41055427
AT83	*Escherichia coli*	MDR clinical isolate from urine culture a 66-year-old woman with urinary tract infection	This study
AT84	*Escherichia coli*	MDR clinical isolate from urine culture a 54-year-old man with urinary tract infection	PMID: 41055427
AT85	*Escherichia coli*	MDR clinical isolate from urine culture a 73-year-old woman with urinary tract infection	This study

AT86	*Escherichia coli*	MDR clinical isolate from urine culture a 63-year-old woman with urinary tract infection	This study

Table S2. Minimum inhibitory concentrations (MICs) of antibiotics against clinically isolated UPEC. Minimum inhibitory concentrations (MICs) of amikacin, ceftriaxone, ciprofloxacin, colistin, gentamicin, and meropenem were determined and are presented in µg/mL. Antimicrobial susceptibility was interpreted according to CLSI breakpoints and is indicated in parentheses as susceptible (S), intermediate (I), or resistant (R). The MDR-UPEC strains were highlighted in a grey label.

Strains	Antibiotics					
	Amikacin	Ceftriazone	Ciprofloxacin	Colistin	Gentamicin	Meropenem
AT1	1 (S)	1 (S)	0.06 (S)	4 (R)	32 (R)	0.6 (S)
AT2	1 (S)	16 (R)	8 (R)	1 (I)	1 (S)	0.6 (S)
AT3	1 (S)	1 (S)	0.06 (S)	1 (I)	1 (S)	0.6 (S)
AT4	1 (S)	1 (S)	0.06 (S)	1 (I)	1 (S)	0.6 (S)
AT5	4 (S)	32 (R)	8 (R)	1 (I)	1 (S)	0.6 (S)
AT6	1 (S)	1 (S)	0.06 (S)	1 (I)	0.25 (S)	0.6 (S)
AT71	1 (S)	1 (S)	1 (R)	8 (R)	2 (S)	0.6 (S)
AT72	1 (S)	64 (R)	0.06 (S)	8 (R)	32 (R)	0.6 (S)
AT73	1 (S)	64 (R)	0.06 (S)	8 (R)	1 (S)	0.6 (S)
AT79	1 (S)	1 (S)	8 (R)	1 (I)	1 (S)	0.6 (S)
AT80	1 (S)	1 (S)	0.06 (S)	1 (I)	1 (S)	0.6 (S)
AT81	1 (S)	4 (R)	8 (R)	1 (I)	1 (S)	0.6 (S)
AT82	1 (S)	64 (R)	1 (R)	1 (I)	32 (R)	0.6 (S)
AT83	1 (S)	1 (S)	0.06 (S)	1 (I)	1 (S)	0.6 (S)
AT84	1 (S)	64 (R)	1 (R)	1 (I)	32 (R)	0.6 (S)
AT85	1 (S)	1 (S)	0.06 (S)	1 (I)	32 (R)	0.6 (S)
AT86	1 (S)	1 (S)	0.06 (S)	1 (I)	1 (S)	0.6 (S)

Table S3. List of qPCR primers used in this research

Species	Gene	Sequence (5’->3’)	Reference
Homo sapiens	*GAPDH*	CCAGGAAATGAGCTTGACAAAGT	PMID: 15908363
		CCCACTCCTCCACCTTTGAC	
	IL8	GCCAACACAGAAATTATTGTAAAGCTT	PMID: 12390316
		CCTCTGCACCCAGTTTTCCTT	
	MIP-3 alpha	CTGCTTTGATGTCAGTGCTGCTAC	PMID: 16714574
		CTGCCGTGTGAAGCCCACAATAAA	
	IL-1beta	AAACAGATGAAGTGCTCCTTCCAGG	PMID: 7814646
		TGGAGAACACCACTTGTTGCTCCA	
	IL-6	ATGAACTCCTTCTCCACAAGCGC	
		GAAGAGCCCTCAGGCTGGACTG	
	TNF-alpha	CGGGACGTGGAGCTGGCCGAGGAG	
		CACCAGCTGGTTATCTCTCAGCTC	
